# The economic burden of Type 2 Diabetes by social determinants of health: A systematic review

**DOI:** 10.1371/journal.pone.0354198

**Published:** 2026-07-27

**Authors:** Dulce E. Alarcón-Yaquetto, Robert Stewart, Khalida Ismail, James Shearer

**Affiliations:** 1 Health Service and Population Research, Institute of Psychiatry, Psychology and Neuroscience, King’s College London, London, United Kingdom; 2 Institute of Psychiatry, Psychology and Neuroscience, King’s College London, London, United Kingdom; 3 South London and Maudsley NHS Foundation Trust, London, United Kingdom; Ministry of Health, Sri Lanka, SRI LANKA

## Abstract

**Background:**

The unequal distribution of resources in society generates social gradients that translate into health inequalities and differential use of health care resources and their costs. Non-medical factors such as employment, income, ethnicity and education impact the prevalence and treatment outcomes of patients with type 2 diabetes mellitus (T2DM); however, there is a scarcity of articles assessing the relationship between health inequalities and the economic costs of treatment. Therefore, we conducted a systematic review of published studies examining the cost differences of treating T2DM across social determinants of health (SDH).

**Methods:**

We systematically searched MEDLINE, Embase, PsycINFO, EconLit, and NHS EED for original peer-reviewed articles that provided cost differences of treating T2DM by SDH: education, income, employment, residency and ethnicity. We grouped the studies by each SDH and calculated the percentage differences where possible between the lowest and highest ends of the gradient (education, income and employment). Residency was categorised as rural vs. urban and ethnicity as white or general population vs other ethnic minorities.

**Results:**

We included 19 articles retrieved internationally from varying healthcare systems. Results were contextualised given the healthcare financing model. In countries with high out-of-pocket expenses, Black and Hispanic ethnic backgrounds and rural residence were associated with lower direct health care and costs likely to be determined by ability to pay rather than clinical need. Indirect costs such as lost productivity due to absenteeism were also lower in unemployed, and lower income groups.

**Conclusions:**

There are evident health disparities in the direct and indirect economic consequences of T2DM. The effect of decreased healthcare use and costs on treatment outcomes needs to be further explored to inform policies to ensure healthcare delivery is based on clinical need rather than socio-economic factors.

## Introduction

Inequality describes the uneven distribution of material resources or opportunities within society. Social variables such as gender, ethnicity, education, and income, among others, shape these opportunities and create a gradient of inequality [1,2]. The impact of inequality on health has been widely documented [3] and there is consensus that social, non-medical factors can determine health outcomes, thus referred to as social determinants of health (SDH) [4].

Chronic diseases —where lifestyle plays a strong role in aetiology and recovery— are greatly affected by SDH [5]. Unhealthy lifestyles are often grouped into clusters associated with social structures and living conditions that restrict the availability and accessibility of healthy choices. These social structures are generally marked by differences in gender, ethnicity, income, and social position [6].

There is ample evidence on the impact of SDH on type 2 diabetes (T2DM) prevalence and complications. For example, a systematic review showed that economic inequalities affect the number of complications in people with T2DM [7], and glycaemic control was also found to be affected by SDH in varying degrees [8], while an interaction between socioeconomic deprivation and genetic predisposition might explain the increased prevalence of the condition in people from South Asian and African backgrounds [9]. Therefore, addressing inequalities is emphasised as means to reduce T2DM incidence and improve health outcomes of people with T2DM [10].

T2DM was selected as the focus of this review because it is among the most prevalent chronic conditions globally and one of the most strongly socially patterned, with its aetiology, management, and complications associated to modifiable lifestyle factors that are themselves shaped by SDH [5,6]. T2DM management is largely ambulatory, requiring sustained engagement with primary care, medication adherence, dietary change, and self-monitoring, which makes it sensitive to the access and resource barriers that vary across the social gradient. T2DM is a significant driver of healthcare expense around the world. Direct costs which encompass the expenses associated with the diagnosis, treatment, and management of the condition are projected to cost 1.51 trillion dollars worldwide by 2030. Indirect costs such as productivity losses due to absenteeism or premature death will reach 0.73 trillion dollars globally according to the same estimation [11]. In addition, intangible costs —those related to the burden of living with T2DM— have also been estimated although less frequently as they are much harder to assess. For instance, one study estimated these at $497 (2019 USD) per person per year in a developing country [12].

SDH also determine health service use, although the direction is not universal and is often country specific. For example, in the UK, a steep gradient in inpatient hospital admissions associated with neighbourhood deprivation has been reported. The costs were estimated at £4.8 billion a year which were partially offset by the lower life expectancy of the most deprived groups [13]. While in Sweden, even after adjusting for age and sex, the highest income groups had increased per capita health expenditures [14]. However, in general, the costs of T2DM according to SDH have not been thoroughly assessed.

Given that T2DM imposes a significant financial impact on healthcare systems and this is potentially greatly affected by SDH, this review aimed to analyse published evidence on the economic burden of health disparities as defined by SDH in the treatment of people with T2DM.

While the impact of SDH on T2DM prevalence, complications, and glycaemic control has been systematically reviewed, the economic costs of T2DM according to SDH have not been synthesised. To the best of our knowledge, this is the first systematic review to synthesise the economic costs of T2DM by SDH. Beyond documenting these costs, this review identifies that the direction of cost inequality is moderated by healthcare system structure, generating a conceptual framework that may have broader applicability to chronic disease cost research.

## Methods

### Search strategy

The protocol of the review was registered on PROSPERO (code CRD42023444818) and is available at https://osf.io/w9nku/files/n9j34. We followed the Preferred Reporting Items for Systematic Reviews and Meta-Analyses (PRISMA) reporting guidelines [15]. The Medical subject headings (MeSH) terms used included: type 2 diabetes, non-insulin dependent diabetes, social determinants of health, health inequalities, healthcare disparities, socioeconomic factors, cost of illness, healthcare costs and hospital costs. The complete search strategy and PRISMA checklist are presented in supplementary file 1 and 2 respectively.

### Study selection and data extraction

We included observational studies (cross-sectional, cohort, and case-control designs) reporting costs of type 2 diabetes mellitus in relation to social determinants of health. Interventional studies, including randomised controlled trials, cost-effectiveness analyses, simulation studies, and economic modelling studies, were excluded. No restrictions were applied based on country or language. All searches were limited to human studies. We also excluded book chapters, communications, editorials, and conference abstracts.

The search took place on 2^nd^ of June 2023. An update was performed the 5^th^ of October 2025. The following databases were searched: MEDLINE, Embase, PsycINFO, EconLit, and NHSeed. Extracted data included country, design, sample size, economic approach, and perspective as well as SDH assessed and cost components. Data was extracted by DEAY from tables, text, or graphs from the original articles. We also evaluated the type of healthcare system in which each study was conducted to contextualise and discuss the results more effectively.Extracted data was compiled in a Microsoft Excel spreadsheet and managed with Mendeley v1.19.8.

Titles and abstracts were reviewed by two researchers (DEAY and JS). When there were divergences, the full text was retrieved. If there was hesitation about the inclusion of a study, the reviewers discussed until a consensus was reached.

The outcome of interest were direct, indirect or intangible costs of T2DM by social determinants of health (SDH). As per their definition, SDH are shaped by political, social and economic forces and are heavily influenced by the distribution of resources. Although the gradient and nature of SDH vary across countries, negative determinants tend to cluster in poorer countries, leading to greater inequities in SDH and, consequently, to health inequalities [16]. For this reason, in the present study, our primary outcome is SDH, used as a proxy for inequality. All included studies reported costs associated with at least one SDH. Residency, education, employment, income, and ethnicity were included as SDH. Gender was excluded as most studies on healthcare costs tend to focus on biological sex rather than gender, which refers to socially constructed norms shaping lifestyles and behaviours. Biological sex, while associated with differences in T2DM prevalence and onset of complications through the protective effect of estrogen [17,18],. is a biomedical rather than social determinant and falls outside the SDH framework. Gender norms may influence cost-relevant behaviours such as health-seeking and treatment adherence. In keeping with the definition of SDH, which refers specifically to non-medical factors, we chose not to include biological sex in our analysis.

### Data synthesis

To allow a better comparison of cost differences, we calculated the percentage difference in each of the studies and grouped the results by SDH. We present these results as comparisons between the lowest and highest categories of each social determinant and by type of costs (direct, indirect, intangible). We took this approach as the operationalisation of each SDH varied by study.

For education and income, percentage differences were calculated between the ones at the lowest education/income category vs those at the highest. For employment, we provide percentage differences between unemployed vs employed. For residence, we compared rural vs urban. For ethnicity and/or race, terms used interchangeably across included studies, we compared ethnic or racial minority groups against white populations or the general population, as defined by each study. The conflation of race and ethnicity across studies represents a source of heterogeneity, as racial categories (i.e., Black, White) reflect socially constructed classifications based on perceived physical characteristics, while ethnic categories reflect shared cultural or ancestral identity [19,20]. Where studies explicitly used racial rather than ethnic categories, we retained the original authors’ terminology. Percentage differences represent the relative difference in costs for the disadvantaged group compared with the reference group. For example, a reported percentage difference for lower versus higher educational attainment indicates how much higher or lower the costs were for less educated individuals relative to those with the highest level of education.

Percentage differences have limitations as a comparative metric when SDH categories are defined inconsistently across studies. For example, ‘low income’ may refer to the bottom quartile in one study and below a specified amount in another, meaning that percentage differences reflect within-study gradients rather than directly comparable cross-study magnitudes. Therefore, these figures should be taken with caution and as indicative of the direction and approximate scale of cost inequalities within each SDH category rather than comparable estimates.

Some studies did not provide a statistical analysis of their results, or only provide crude or adjusted p-values. We provide both values when available and report if adjusted or crude p-values were not given. In text, any non-significant result means the p value was above 0.05. The results are presented graphically. Due to the high heterogeneity of the retrieved studies, a meta-analysis was not feasible.

### Quality assessment

The quality was assessed using Larg and Moss tool for cost-of-illness studies [21] which analyses three main domains: analytical framework, methodology and data, and analysis and reporting. Each with subdomains that allow a comprehensive appraisal of the study such as the perspective, epidemiological approach, and the methodology used to quantify the costs. Each item was rated as low risk of bias, high risk of bias, unclear or no information. Quality appraisal was conducted by one reviewer (DEA-Y). Where uncertainty arose regarding the classification of individual items, assessments were discussed with a second reviewer (JS) to reach consensus. The results are presented graphically using *robvis* package [22] in RStudio.

## Results

### Included studies

**Fig 1:**
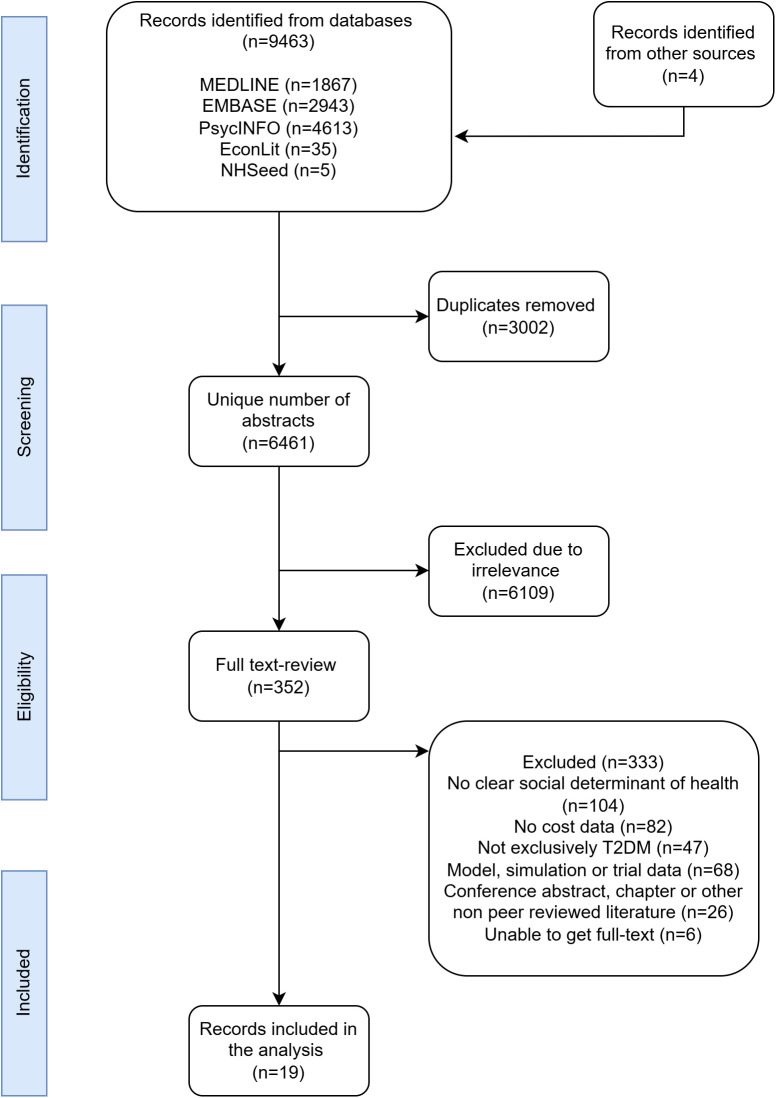
PRISMA flowchart.

The initial search yielded 5819 unique studies of which 5488 were excluded as they were deemed irrelevant to the research question, 331 studies underwent full review and 22 were selected for analysis. An updated search conducted in October 2025 identified an additional 642 records. Of these, 21 studies were assessed at the full-text stage; however, none met the inclusion criteria, and no additional studies were included in the final analysis. The complete study selection process is illustrated in Fig 1.

The median sample size was 10575 (range: 1021−32,846). Most studies had a cross-sectional design (68%), and publications dates ranged from 2000 to 2021. Table 1 presents further characteristics of the studies analysed.

**Table 1:** Characteristics of the selected studies.

Article	Country	Design	Sample size	Data source	Approach	Perspective	Country’s healthcare model	Reference
**Afroz 2019**	Bangladesh	Cross-sectional	1253	Hospital records and questionnaires	Prevalence	Patients	Out-of-pocket	[23]
**Le 2013**	China	Cross-sectional	9396	Community health interview and survey	Prevalence	Societal	Out-of-pocket	[24]
**Lee 2006**	USA	Cross-sectional	1021	Survey	NR	NR	Mixed	[25]
**Li 2015**	USA	Prospective	11927	Survey, medical records and administrative data	NR	HC	Mixed	[26]
**Daniel 2021**	India	Prospective	300	Medical bills, records and questionnaires	NR	NR	Out-of-pocket	[27]
**Dawson 2021**	USA	Cross-sectional	17820243	Survey	NR	NR	Mixed	[28]
**Endarti 2020**	Indonesia	Cross-sectional	131	Questionnaires	Prevalence	Patients	Mixed	[29]
**Guo 2021**	China	Population based survey	17708	Survey	NR	Societal	Out-of-pocket	[30]
**Hazel Fernández 2015**	USA	Retrospective	333576	Medical claims	NR	NR	Mixed	[31]
**Hu 2015**	USA	Longitudinal	32846	Survey	NR	NR	Mixed	[32]
**Jacobs 2000**	Canada	Cross-sectional	894420	Healthcare records and registries	NR	NR	National Health Insurance	[33]
**Jing 2019**	China	Cross-sectional	1948	Questionnaire	NR	NR	Out-of-pocket	[34]
**Kim 2011**	Korea	Cross-sectional	2752	Hospital electronic databases and questionnaires	NR	NR	National Health Insurance	[35]
**Konig 2021**	Germany	Cross-sectional	325	Survey	NR	Societal	Insurance based	[36]
**Kumar 2008**	India	Cross sectional	1153	Survey	NR	NR	Out-of-pocket	[37]
**Khowaja 2007**	Pakistan	Cross-sectional	353	Questionnaire	Prevalence	Societal	Out-of-pocket	[38]
**Simmons 2019**	USA	Cross-sectional	17702	Survey	NR	NR	Mixed	[39]
**Sun 2018**	China	Cross-sectional	661	Questionnaire	NR	Societal	Out-of-pocket	[40]
**Williams 2020**	USA	Longitudinal	11755	Survey	NR	NR	Mixed	[41]

NR: Not reported, NA: Not applicable.

Twelve studies evaluated only direct costs [25,26,28,30–35,37,39,41], while seven evaluated both [23,24,27,29,36,38,40]. One study from the latter group also evaluated intangible costs [24]. Some studies further classified direct costs as medical and non-medical costs. The former refers to all medical procedures and physician fees associated with medical treatment while the non-medical category includes cost of transportation, accommodation and meals while traveling to the healthcare centre. Table 2 provides detailed information of what costs and components were analysed in each study and the cost allocation method used.

**Table 2:** Types of costs, components and costing methods used in included studies.

Article	Type of direct costs	Cost allocation direct costs	Type of indirect cost	Cost allocation indirect costs	Intangibles	Counterfactual	SDH analysed
**Afroz 2019**	Direct medical (hospitalisation, outpatient visits, medicine, laboratory tests, glucose monitoring, laboratory tests and consumables) Direct non-medical (transportation and meals to hospital)	Bottom-up (micro-costing)	Patients and carers’ productivity	Human capital approach	NA	None	Residence, Education, Employment, Income
**Le 2013**	Direct medical (hospitalisation, outpatient visits, and medication) Direct non-medical costs (transportation for the patient and carers to and from healthcare providers, accommodation, and costs of hiring nurses or other care providers)	NR	Productivity loss due to premature mortality and productivity loss related morbidity	Human capital approach	Contingence valuation willingness to pay approach on losses due to physical and psychological complications	None	Education
**Lee 2006**	Direct medical (ambulatory care visits and prescription drug fills). Disaggregated in payments by insurance and OOP	NR	NA	NA	NA	None. Comparisons made between T2DM patients by ethnicity	Ethnicity
**Li 2015**	Direct medical (inpatient, outpatient, emergency, pharmacy, radiology and laboratory tests)	NR	NA	NA	None	None	Ethnicity, Education, Income
**Daniel 2021**	Direct medical (hospital services, physician services, laboratory tests, daily management, medicines, consultations, hospitalisations)	NR	Loss of productivity due to sickness. Monthly income loss of patient and carer. Transportation and other ancillary costs were considered indirect costs.	Daily wages multiplied by number of absent days		None. T2DM patients grouped between rural vs urban	Residence
**Dawson 2021**	Direct medical (office-based medical provider expenditure, outpatient, inpatients, emergency, prescriptions, dental expenditure, home health care expenditure) whether OOP or paid by insurance provider	NR	NA	NA	NA	None	Ethnicity
**Endarti 2020**	Direct medical costs (administration, healthcare professionals, laboratory test cost, drug and medical item costs). Direct non-medical (meal and transportation of patients and caregivers)	NR	Productivity loss of patients and caregivers	Human capital approach	NA	None	Employment, Income
**Guo 2021**	Direct medical costs (medications and tests, outpatients, inpatients visits, and self-health care). Direct nonmedical costs (transportation, visits by relatives and/or friends during inpatient and outpatient periods, accommodations, and food)	NR	NA	NA	NA	None. Population was divided in patients with diagnosed T2DM and those with undiagnosed T2DM. Costs extracted refer to diagnosed T2DM	Residence, Education, Employment, Income
**Hazel Fernández 2015**	Direct medical costs (all-cause medical and pharmacy claims, irrespective of diagnosis or therapeutic class for the respective medications, outpatient and inpatient hospitalisation use, emergency)	NR	NA	NA	NA	None. Comparisons made between T2DM patients by ethnicity	Ethnicity
**Hu 2015**	Direct medical costs (medical care, hospital use, physician office visits, and prescription drug use) whether OOP or paid by insurance provider	NR	NA	NA	NA	Yes. Non-diabetes group	Education, Employment, Income, Ethnicity
**Jacobs 2000**	Direct medical costs (physician fees, personal care home days, hospitalisation, outpatient dialysis)	NR	NA	NA	NA	Yes. Population was grouped in First Nation and General Population. Each group was subdivided in those with or without T2DM	Ethnicity
**Jing 2019**	Direct medical costs (outpatient, inpatient, physical examinations)	NR	NA	NA	NA	None	Residence
**Kim 2011**	Direct medical costs (diagnosis, outpatients, hospitalisation, pharmacological therapy and cost of complementary alternative medicine)	NR	NA	NA	NA	None. T2DM population was grouped between CAM users vs non users. Cost provided in the analysis belong to non-users.	Education, Income,
**Konig 2021**	Direct (outpatient, inpatient, rehabilitation and medication)	Bottom-up	Sick leave and early retirement	Human capital approach	NA	Yes. Patients with T2DM and non-T2DM	Education,
**Kumar 2008**	Direct medical (consultations, medicines, tests, monitoring)	NR	NA	NA	NA	No	Education, Employment, Income,
**Khowaja 2007**	Direct medical (consultations, investigations, medicine) Direct non-medical (travel and food)	Top down	Loss productivity because of disability and premature death and loss of time (travelling, waiting, getting healthcare) of patients and carers	Human capital approach	NA	No	Education, Income
**Simmons 2019**	Direct medical OOP (medical provider, non-physician services, inpatients, emergency, dental care, medications)	NR	NA	NA	NA	T2DM sample divided by ethnicity. No counterfactual.	Ethnicity
**Sun 2018**	Direct medical (outpatient, inpatient, drugs, medical equipment) Direct non-medical (transportation, accommodation, costs providing nutritional food according to special needs of T2DM patients)	NR	Productivity loss of patients and carers	Lost earnings due to lost workdays		T2DM population divided by Hui and Han	Ethnicity
**Williams 2020**	Direct medical (healthcare, hospital and emergency visits, pharmacy and home-health care)	NR	NA	NA	NA	T2DM only women patients were divided by ethnicity	Ethnicity

OOP: Out-of-pocket, T2DM: Type 2 diabetes mellitus, CAM: complementary alternative medicine, NA: Not applicable, NR: Not reported.

### Healthcare models

Seven studies were set in the US, which has a mixed healthcare model with low level of government intervention [42]. In 2020, 9% of the US population were not covered by any health insurance. Private health insurance coverage was more prevalent than public coverage at 67% and 35%, respectively [43]. In 2022, 11% of US health care expenditures were paid out of patients’ pockets directly or through deductibles [44].

Four studies were set in China, where the healthcare system has undergone major reforms in recent years aiming for universal coverage and decreasing catastrophic OOP expenditure [45]. Now, citizens are enrolled either in the employee basic medical insurance programme or the non-working residents’ scheme which also serves rural populations [46]. However, in 2016, 29% of Chinese residents still incurred healthcare OOP expenses [45].

Countries in South Asia (Bangladesh, India, Pakistan) were also represented in the selected studies. These are examples of countries where the patient is the main bearer of costs. In Bangladesh, 64% of overall healthcare expenditure is OOP [47], 63% in India [48] and 88% in the private sector in Pakistan [49].

In Indonesia, where one study was conducted, the healthcare systems is fragmented. Until 2014, Indonesia had employment-based insurance schemes, public assistance services for the uninsured, and a private sector, until a reform scheme came into place that sought to unify multiple fragmented programmes, it now covers 83% of their population [50].

Canada and South Korea, with one study each, have a National Health Insurance model where the providers are private, and the funds are public [51,52]. The coverage of basic medical and hospital costs is complete and in Canada, without direct charges at the point of delivery. In Korea, patients pay 30% of outpatient medical and prescriptions costs [53]. Finally, Germany (one study) has an insurance system financed by employers and employees through payroll deduction. Insurance plans do not make profit and include all citizens [54]. It is possible to opt out of the insurance scheme in certain cases, such as being a high-earner [55].

### Primary outcomes

Findings were conflicting for education, income, and employment, while residence, and ethnicity showed clearer and more sustained trends across studies.

#### Residence.

**Fig 2:**
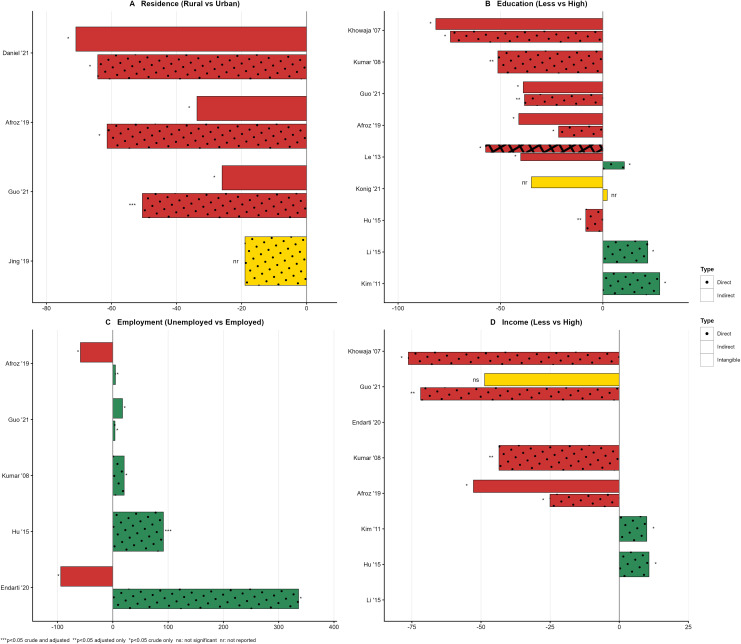
Percentage difference according to (A) Residence status, (B) Education, (C) Employment and (D) Income.

Residence status was ascertained by urban or rural settings in 4 studies, all set in the Asian continent [23,27,30,34]. All four studies reported that the direct cost of treating T2DM in rural settings was lower than the treatment of urban patients. Two studies also reported decreased indirect costs [23,27]. The differences ranged from 18% [34] to 71% [27] (Fig 2A). Out-of-pocket (OOP) expenditure was reported in three studies as part of direct medical costs [24,27,30,34]. Guo reported OOP being higher in rural areas [30], Daniel reported higher catastrophic OOP costs in urban areas [27] and Jing reported no difference in OOP costs but the proportion of the OOP relative to their total income was higher for rural patients [34].

#### Education.

Data regarding education was mixed. Nine studies provided costs by this SDH. Five provided only direct costs, and four provided both. Additionally, one study from the latter group also provided intangible costs [24]. Regarding direct costs, three studies reported that people with lower educational attainment had higher costs than their higher educated counterparts [24,26,35]. Only crude p values were reported. On the other hand, five studies reported decreased direct costs in people with lower educational level [23,30,32,37,38]. Some studies reported significant differences even after adjusting by other social variables and comorbidities [30,32,37] and pharmacological adherence [37] (Fig 2B).

Intangible costs, defined as the willingness to pay to avoid the physical and psychological strain of T2DM, were 41% lower for less educated people [24] (Table 3, Fig 2B). Four studies reported that lower education was associated with decreased indirect costs, mainly assessed by the loss of productivity of the patient and their carers due to the disease [23,24,36,38]. Three reported significant differences in crude models while one reported 35% less indirect costs but does not provide statistical testing.

**Table 3:** Summary of percentage cost differences in the treatment of type 2 diabetes by social determinant of health, grouped by region and healthcare model.

SDH	Region	Healthcare model	Study	SDH operationalisation	Cost type	% difference	Direction
**Residence**	Asia	OOP	Daniel 2021	Rural vs Urban	Direct	64.23% *, nr	↓ Rural
**Residence**	Asia	OOP	Afroz 2019	Rural vs Urban	Direct	61.36% *, nr	↓ Rural
**Residence**	Asia	Mixed/OOP	Jing 2019	Rural vs Urban	Direct	18.95% nr	
**Residence**	Asia	Mixed/OOP	Guo 2021	Rural vs Urban	Direct	50.52% *, *	↓ Rural
**Residence**	Asia	OOP	Daniel 2021	Rural vs Urban	Indirect	70.95% *, nr	↓ Rural
**Residence**	Asia	OOP	Afroz 2019	Rural vs Urban	Indirect	33.72% *, nr	↓ Rural
**Residence**	Asia	Mixed/OOP	Guo 2021	Rural vs Urban	Indirect	26% *, ns	↓ Rural
**Education**	USA	Mixed	Li 2015	Less than high school vs completed college	Direct	21.95% *, nr	↑ Less than high school
**Education**	USA	Mixed	Hu 2015	No degree vs above Bachelor	Direct	8.35% ns, *	↓ No degree
**Education**	Asia	OOP	Afroz 2019	Illiterate vs Tertiary	Direct	21.61% *, nr	↓ Illiterate
**Education**	Asia	Mixed/OOP	Le 2013	Illiterate vs higher than middle school	Direct	10.52% *, nr	↑ Illiterate
**Education**	Asia	Mixed/OOP	Kumar 2008	Illiterate vs Postgraduate	Direct	51.26% nr,*	↓ Illiterate
**Education**	Asia	Mixed/OOP	Khowaja 2007	Illiterate vs Higher than secondary	Direct	74.46% *, nr	↓ Illiterate
**Education**	Asia	Mixed/OOP	Guo 2021	Primary vs high school	Direct	38.28% ns,*	↓ Illiterate
**Education**	Asia	OOP	Afroz 2019	Illiterate vs Tertiary	Indirect	41.05% *, nr	↓Illiterate
**Education**	Asia	Mixed/OOP	Le 2013	Illiterate vs higher than middle school	Indirect	40.12% *, nr	↓ Illiterate
**Education**	Asia	Mixed/OOP	Khowaja 2007	Illiterate vs Higher than secondary	Indirect	81.56% *, nr	↓ Illiterate
**Education**	Asia	Mixed/OOP	Guo 2021	Primary vs high school	Indirect	38.88% *, ns	↓ Illiterate
**Education**	Asia	Mixed/OOP	Le 2013	Illiterate vs higher than middle school	Intangible	57.25% *, nr	↓ Illiterate
**Education**	Asia	National Health Insurance	Kim 2011	Less than high school vs More than High School	Direct	27.8% *, nr	↑Less than High School
**Education**	Europe	Insurance based	Konig 2021	Low vs High	Direct	2.14% nr	
**Education**	Europe	Insurance based	Konig 2021	Low vs High	Indirect	34.88% nr	
**Employment**	Asia	Mixed/OOP	Kumar 2008	Unemployed vs Employed	Direct	20.58% *, ns	↑ Unemployed
**Employment**	Asia	Mixed/OOP	Guo 2021	Unemployed vs Employed	Direct	3.95% *, ns	↑ Unemployed
**Employment**	Asia	Mixed	Endarti 2020	Unemployed vs Employed	Direct	336% *, nr	↑ Unemployed
**Employment**	Asia	OOP	Afroz 2019	Unemployed vs Employed	Direct	4.78% *, nr	↑ Unemployed
**Employment**	USA	Mixed	Hu 2015	Unemployed vs Employed	Direct	91.62% *,*	↑ Unemployed
**Employment**	Asia	Mixed	Endarti 2020	Unemployed vs Employed	Indirect	94.17% *, nr	↓ Unemployed
**Employment**	Asia	OOP	Afroz 2019	Unemployed vs Employed	Indirect	58.62% *, nr	↓ Unemployed
**Employment**	Asia	Mixed/OOP	Guo 2021	Unemployed vs Employed	Indirect	17.6% *, ns	↑ Unemployed
**Income**	Asia	OOP	Afroz 2019	MHI < US$250 vs US$751+	Direct	25.05% *, nr	↓ MHI < US$250
**Income**	Asia	Mixed	Endarti 2020	<3,000,000 vs ≥ 3,000,000	Direct	161.43% ns, nr	↓ < 3,000,000
**Income**	Asia	Mixed/OOP	Guo 2021	Lowest 20% vs Highest 20%	Direct	71.86% ns, *	↓ Lowest 20%
**Income**	Asia	Mixed/OOP	Kumar 2008	YI < 0.36 million INR vs > 1.2 million	Direct	43.5% nr,*	↓ YI < 0.36 million INR
**Income**	Asia	Mixed/OOP	Khowaja 2007	MHI < 5000 PKR vs > 20000 PKR	Direct	76.29% *, ns	↓ MHI < 5000 PKR
**Income**	Asia	National Health Insurance	Kim 2011	MHI US$ < 2000 vs > 40000	Direct	9.98% *, nr	↑ MHI US$ < 2000
**Income**	USA	Mixed	Li 2015	YI < $15000 vs > 74999	Direct	59.24% ns, nr	
**Income**	USA	Mixed	Hu 2015	<US$20000 vs>$40000	Direct	10.77% *,ns	↑ < US$20000
**Income**	Asia	Mixed/OOP	Khowaja 2007	MHI < 5000 PKR vs > 20000 PKR	Indirect	84.48% *, ns	↓ MHI < 5000 PKR
**Income**	Asia	Mixed	Endarti 2020	<3,000,000 vs ≥ 3,000,000	Indirect	65.08% *, nr	↑ < 3,000,000
**Income**	Asia	Mixed/OOP	Guo 2021	Lowest 20% vs Highest 20%	Indirect	48.68% ns, ns	
**Income**	Asia	OOP	Afroz 2019	MHI < US$250 vs US$751+	Indirect	52.75% *, nr	↓ MHI < US$250
**Ethnicity**	Canada	National Health Insurance	Jacobs 2000	1st nation vs general	Direct	68.56% (nr)	
**Ethnicity**	Asia	Mixed/OOP	Sun 2018	Hui vs Han	Direct	26.65% (*, *)	↓ Hui
**Ethnicity**	Asia	Mixed/OOP	Sun 2018	Hui vs Han	Indirect	74.92% (*, *)	↓ Hui
**Ethnicity**	USA	Mixed	Dawson 2021	Hispanic vs Non Hispanic White	Direct	32.54% (nr, *)	↓ Hispanic
**Ethnicity**	USA	Mixed	Hazel-Fernandez 2015	Hispanic vs Non Hispanic White	Direct	10.66% (*, nr)	↓ Hispanic
**Ethnicity**	USA	Mixed	Hu 2015	Hispanic vs Non Hispanic White	Direct	34.21% (*, *)	↓ Hispanic
**Ethnicity**	USA	Mixed	Lee 2006	Hispanic vs Non Hispanic White	Direct	18% (ns, *)	↓ Hispanic
**Ethnicity**	USA	Mixed	Li 2015	Hispanic vs Non Hispanic White	Direct	26.21% (ns,*)	↓ Hispanic
**Ethnicity**	USA	Mixed	Simmons 2019	Hispanic vs Non Hispanic White	Direct	38.38% (ns,*)	↓ Hispanic
**Ethnicity**	USA	Mixed	Williams 2020	Hispanic vs Non Hispanic White	Direct	41.66% (*, *)	↓ Hispanic
**Ethnicity**	USA	Mixed	Dawson 2021	Non Hispanic Black vs Non Hispanic White	Direct	4.42% (nr, ns)	
**Ethnicity**	USA	Mixed	Hazel-Fernandez 2015	Non Hispanic Black vs Non Hispanic White	Direct	3.02% (*, nr)	↓ NHB
**Ethnicity**	USA	Mixed	Hu 2015	Non Hispanic Black vs Non Hispanic White	Direct	0.69% (*,ns)	
**Ethnicity**	USA	Mixed	Lee 2006	Non Hispanic Black vs Non Hispanic White	Direct	10.52% (ns, *)	↓ NHB
**Ethnicity**	USA	Mixed	Li 2015	Non Hispanic Black vs Non Hispanic White	Direct	15.59% (ns, *)	↓ NHB
**Ethnicity**	USA	Mixed	Simmons 2019	Non Hispanic Black vs Non Hispanic White	Direct	26.62% (ns, *)	↓ NHB
**Ethnicity**	USA	Mixed	Williams 2020	Non Hispanic Black vs Non Hispanic White	Direct	11.75% (ns,*)	↓ NHB
**Ethnicity**	USA	Mixed	Dawson 2021	Other vs Non Hispanic White	Direct	33.61% (nr, *)	↓ Other
**Ethnicity**	USA	Mixed	Hazel-Fernandez 2015	Other vs Non Hispanic White	Direct	21.86% (*, nr)	↓ Other
**Ethnicity**	USA	Mixed	Hu 2015	Other vs Non Hispanic White	Direct	18.65% (*,ns)	↓ Other
**Ethnicity**	USA	Mixed	Li 2015	Other vs Non Hispanic White	Direct	14.67% (ns, *)	↓ Other
**Ethnicity**	USA	Mixed	Simmons 2019	Other vs Non Hispanic White	Direct	21.37% (ns, *)	↓ Other

MHI: Monthly household income, YI: Yearly income. *p < 0.05 When two values are given the 1^st,^ one represents crude p value and the 2^nd^ the adjusted. ns: not significant, nr: not reported.

#### Employment.

Among the studies that reported inequality based on employment status, two found no significant difference in direct costs [30,37]. Direct costs in Bangladeshi patients with T2DM were found to be 5% higher in unemployed patients compared to employed ones (Fig 2C, p < 0.05 in crude analysis) [23]. Interestingly, this study found that housewives and retired patients were those with the highest cost: respectively 21% and 39% higher compared to unemployed patients [23] (p < 0.05 crude between-group comparison). In the US, Hu found significant increased direct costs for the unemployed in a representative sample of adult US patients in both crude and adjusted models [32]. Guo found that retired and self-employed patients had highest direct costs; however, when adjusting the model by other sociodemographic variables, employment was not associated with increased costs [30]. Regarding indirect costs, Endarti *et.al.* and Afroz *et.al.* found that unemployed people had significantly lower indirect costs than employed people [23,29] (Fig 2C).

#### Income.

Eight studies presented cost of T2DM by income (Fig 2D). Four out of the seven studies that reported direct costs found that people with less income incurred lower direct costs [23,30,37,38]. Kim, however, reported that those with less income were associated with significantly higher costs to the universal national health insurance system in Korea [35]. The remaining studies found no significant difference. Regarding indirect costs, there was consistency in reporting lower income associated with significant decreased indirect costs in Bangladesh, Pakistan, and the USA (Fig 2D).

#### Ethnicity.

**Fig 3:**
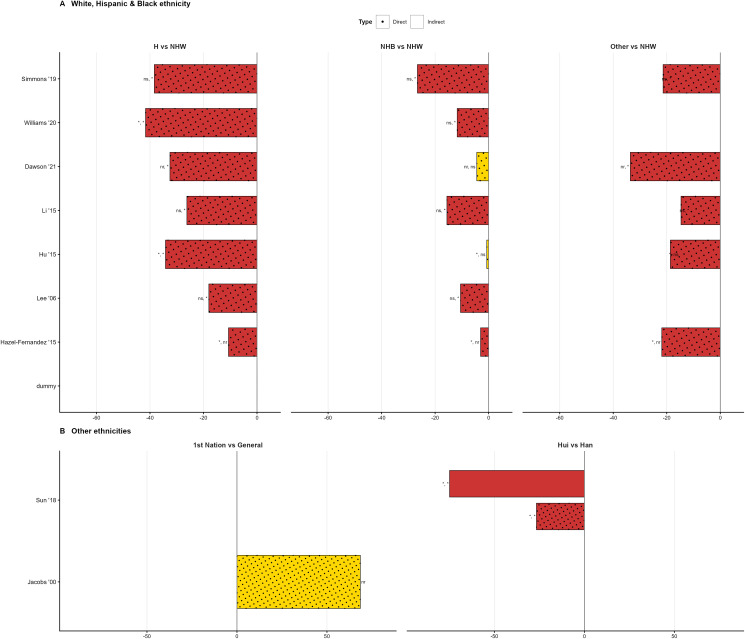
Percentage difference according to ethnicities (A) White, Hispanic and Black ethnicities, (B) Other ethnicities. NHB: Non-Hispanic Black, H: Hispanic, NHW: Non-Hispanic White. *p < 0.05 When two values are given the 1^st,^ one represents crude p value and the 2^nd^ the adjusted. ns: not significant, nr: not reported.

Seven studies set in the US analysed the costs of treating T2DM by ethnicity. The median (p25-p75) sample size of the studies was 16,065 (11,841−183,211). White individuals incurred highest direct healthcare expense compared to Black [25,26,28,31,39,41], Hispanic [25,26,28,31,32,39,41] and other ethnicities [26,28,31,39] (Fig 3A).

Finally, two studies reported costs by ethnic minorities in Canada and in China (Fig 3B). The Canadian study reported that aboriginal First Nation people with T2DM had increased direct healthcare costs compared to non-aboriginal patients [33], with the higher costs driven by 2.74 higher hospital admission rates.

On the other hand, Sun et.al. reported that the Hui ethnic minority in China had significantly lower direct and indirect costs than Han Chinese patients [40]. The indirect costs were explained by the high rates of unemployment and lower income in the Hui while the medical expenditures showed an interesting trend: the authors used quantile regression analysis and found that in the lower quantiles of expense, i.e., healthcare components with lower costs, Hui expenses were significantly lower in crude models but adjusted by education, income and comorbidities, the association was lost. However, in the highest quantile the trend was reversed, and the Hui incurred significantly higher cost than the Han even after adjusting for other variables. Table 3 summarizes these findings and are grouped by region and healthcare model.

### Quality of the studies

**Fig 4:**
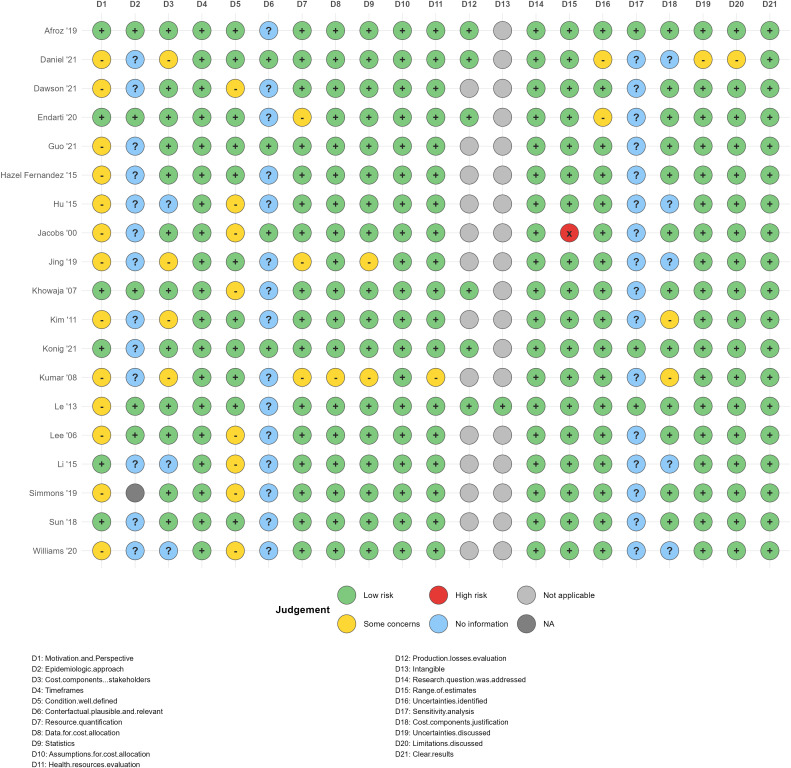
Traffic light plot showing the risk of bias in each domain analysed according to the Larg and Moss appraisal checklist for cost-of-illness studies.

The quality of the studies plot based is presented in Fig 4.

Most of the studies were of high quality, with low risk of bias in domains such as the data for cost allocation or the assumptions made for the quantification of resources. The major concerns arose from a lack of detail on the perspective of the study, with only 8 studies reporting this explicitly. Knowing which perspective is taken is essential to ascertain potential biases that might be triggered if the perspective is only that of the cost-bearer. Furthermore, most studies did not state the epidemiological approach, or perform a sensitivity analysis although this might be due to a lack of standardised guidelines for the reporting of cost studies.

Counterfactuals were missing from 77% of studies, mainly because the purpose of the studies were not to assess the cost attributed to T2DM, but the effect of SDH on costs, whether direct or indirect. A counterfactual is a hypothetical scenario where the study population does not have T2DM but is otherwise identical in all other respects [21]. Instead of analysing a counterfactual (non-T2DM scenario), most studies divided their population based on SDH characteristics. This information is collated in Table 2. We did not subtract quality points if a counterfactual was missing.

## Discussion

The objective of this review was to assess the cost of inequality in the treatment of T2DM. To achieve this, it is essential to discuss the results in the appropriate context. We note that even though we discuss some results considering the healthcare model of the country in which each study was set, it is not the purpose of this article to discuss healthcare financing as a driver of inequalities.

**Fig 5:**
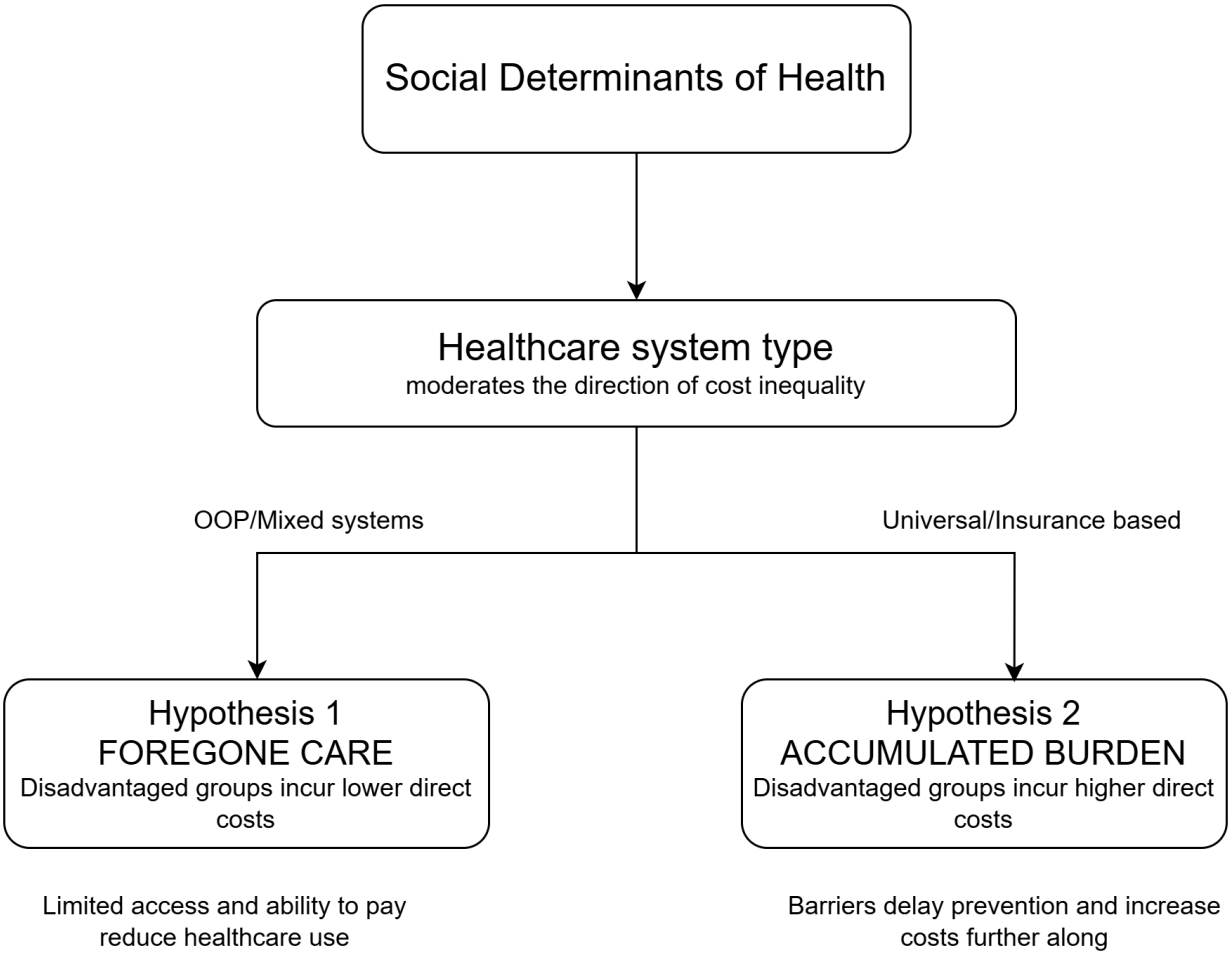
Conceptual framework illustrating how social determinants of health (SDH) influence the economic costs of treating type 2 diabetes mellitus, moderated by healthcare system type.

To interpret the findings of this review, we propose a conceptual framework (Fig 5) comprising two hypotheses that explain how inequalities impact T2DM treatment costs, moderated by each country’s healthcare system structure. These hypotheses are not mutually exclusive and may represent different stages of healthcare engagement among people experiencing inequality.

The first hypothesis: Foregone care: people experiencing inequalities incur lower direct medical costs due to their limited ability to afford the time and resources needed for regular medical visits and treatments. This is supported by articles reporting lower direct costs in rural areas in China, India and Bangladesh where costs are usually paid by the patient. Inequality due to rural residence is linked to lower incomes [56] which in turn only permit uptake of lower-cost treatment options [57]. However, the inequality suffered by rural populations is also experienced in healthcare services and infrastructure which might account for the lower direct costs. Specifically, high-complexity hospitals are not available in Chinese rural areas [58], personnel only work during the daytime in Indian rural healthcare centres [27] and, in Bangladesh and India, healthcare infrastructures and workforce have less provision in rural zones [59–61].

Associations with lower income also support this first hypothesis. In particular, countries where costs were found to be lower in the less well-off have mixed models of healthcare with high OOP. Therefore, the lower cost found is most likely to be associated with poor capacity and/or willingness to pay. Health-care utilization depends on the need for care, awareness of the need, willingness, and accessibility of care [62]. Where patients must bear the cost of a disease, the usage of services might be more associated with capacity and willingness to pay than to clinical needs.

Second hypothesis: Accumulated burden. Inequality leads patients to experience higher direct medical costs in the long run, as inadequate prevention and low access to primary care could lead to the earlier onset of severe complications. While overall results were conflicting, less educated patients tended to have higher direct medical costs: 1) in South Korea, a nation with universal healthcare insurance; 2) in the Chinese rural province of Yunnan, one of the poorest areas of Southwest China with 70% of its population working as farmers, where new rural insurance schemes decrease the odds of patients not seeking healthcare due to capacity to pay; 3) in a US study that analysed patients from a managed care organisation. In the last of these, the sample included patients from 8 different healthcare plans but uninsured patients or those who pay out of pocket were not represented [26]. The increased cost observed in illiterate or less educated patients might reflect lower health literacy, as there is evidence of an association between these factors [63]. Higher health literacy is associated with better health outcomes and a differentiated use of health services. Health literate patients use primary care services more [63] whereas less health literate patients use more emergency services [64] which tend to be costlier. The Chinese and US studies found increased costs but did not provide cost component differentiation. This mediation needs to be thoroughly explored in T2DM for a better understanding of the drivers of allocation of resources.

There were, however, other conflicting results. Less educated patients tended to have lower direct medical costs in countries that do not provide universal healthcare access such as India. Higher educated patients might access more and costlier treatment options than those less educated as they are likely to be in higher paying jobs and have easier access to treatments. In South Korea, the cost of the less deprived were also found to be higher. This might reflect genuine clinical need, increased survival, increased severity of the condition or more complications as the lack of access barrier is gone. In countries with a similar healthcare system to the South Korean model, similar results have been found with health service use and costs increasing with neighbourhood deprivation [13,65].

Inequality due to employment also suggests that those facing joblessness incur greater expense, supporting the second hypothesis. In China and Bangladesh there is a gradient where unemployed people incur higher direct costs than those employed. Retired patients have the highest direct healthcare costs in both studies, although this might be due to age and higher odds of costlier complications and comorbidities. Associations with unemployment might be due to the severity of the disease, which was not ascertained in either study, but which could explain higher direct costs. Leaving the labour market due to disease creates a higher risk for patients who are already in vulnerable sectors of the population such as people with low socioeconomic standing [66]. Patients with T2DM have an increased risk of stopping working prematurely and of experiencing unemployment which imposes costs on the economy as a whole [67]. Regarding employment, it is worth mentioning that some specific occupations have been associated with increased risk of T2DM and its complications such as night-shift workers [68], drivers and cleaners [69] but no studies of costs in these populations were found.

The Indian study showing no significant difference in direct costs according to employment status [37] had a source population of predominantly middle- and high-income patients in Delhi; therefore, the unemployed population was not equivalent to those from China or Bangladesh. In Indonesia, direct medical costs were not associated with employment nor income, but lost productivity was related to income. This country is moving towards a universal healthcare system and T2DM treatment costs are covered [29].

Inequality driven by ethnicity was probably the domain with fewest conflicting results. US based studies that assessed ethnicity all agreed that the white people with T2DM had the highest direct and indirect costs. The effect of ethnicity on diabetic healthcare service use and costs in US ethnic minorities has been attributed to a mixture of economic factors and insurance status. However, some studies adjusted for these variables and still found significant differences which might point out to underlying systemic inequalities such as racism in the provision of care to ethnic minorities.

There is evidence that ethnic minorities have increased odds of therapeutic inertia, including delays in initiating insulin when glycaemic targets are not met [70,71]. As insulin is a much more expensive treatment, underlying issues regarding ethnic differences in this variable need to be addressed as might explain decreased healthcare costs in these demographics. People from Hispanic and Black groups have increased odds of worse T2DM outcomes. The extent of the effect of less healthcare resource use and decreased costs in treatment outcomes needs to be further explored.

Two other ethnic minorities were included in the review with different patterns. The Hui Chinese were compared to the majority Han in China. The Hui have shown to have increased prevalence of metabolic syndrome [72] and cardiovascular disease [73]. This might explain why only in the 90^th^ quintile of costs, the costs are greater than in the Han even after adjusting for confounders. In all other quintiles, the crude cost is lower but is explained by lower education and income which also affects this minority group. Lower cost in lower quintiles might be explained by healthcare foregone owing to delayed healthcare seeking, especially if patients from minority groups must pay for it or have inflexible working hours.

In Canada, the increased costs in the aboriginal minority are potentially explained by the severity of the disease. Besides genetic factors, this population has been generationally isolated and restricted in healthy food choices. Also, their hunting and fishing culture has been impeded which caused a shift in their lifestyle [74]. It is worth noting that, different to the US, in Canada healthcare is free at the point of delivery which might also explain the change of patterns.

These hypotheses may reflect different stages of healthcare utilisation among people experiencing inequalities. The first hypothesis might be more applicable to earlier stages of the condition where preventive treatment is available but underused; while the second hypothesis might take predominance as the condition progresses. Given that studies were mainly cross-sectional, and costs were not ascertained longitudinally it is not feasible to explore this dynamic fully. This highlights the importance of evaluating cost trajectories as well as healthcare resource use alongside costs to get a better understanding where disparities emerge. Therefore, while both hypotheses seem to contradict each other, they might be part of a continuum of healthcare engagement by inequalities that warrants further research.

Regarding indirect costs, it is expected that patients on lower incomes will have lower indirect costs because of worse paid jobs. This is supported by the lower indirect costs found in Bangladeshi rural population as their wages are significantly inferior [75]. Lower indirect costs were also found in unemployed, uninsured and less educated patients. These results are likely driven by fewer missed workdays and the lower cost of each missed workday. Uninsured workers and those with lower educational attainment are likely to be worse paid than those with insurance and will tend to lose fewer workdays because of the higher impact of lost wages. Higher education was associated with higher indirect costs. However, the only study that provided intangible costs, also provided years of life lost due to T2DM whose relationship with educational level is inverse. This highlights that measuring indirect costs, using the human capital approach where income is a main variable or intangible cost measured by the willingness to pay approach, might not provide a thorough picture of the impact of financial costs of T2DM in the life of a patient.

Important policy implications arise from these findings. In countries with high rural OOP burden, expanding rural healthcare infrastructure alone may be insufficient; financial protection mechanisms such as subsidised transport to care or telemedicine reducing travel-related access barriers may be needed to convert foregone care into appropriate utilisation. In universal healthcare systems, the higher costs observed in less educated patients suggest health literacy interventions, particularly around T2DM self-management and primary care engagement, could reduce downstream complication costs. In OOP systems, the same intervention would need to be paired with financial protection, as health literacy alone cannot translate into care-seeking without addressing capacity to pay. As evidence shows a pattern of therapeutic inertia in insulin initiation among ethnic minorities, this becomes a target for quality improvement, monitoring time-to-insulin-initiation by ethnicity within healthcare systems could identify and address a concrete driver of the cost-outcome mismatch identified in this review.

Our review has strengths and limitations. The wide search strategy allowed us to retrieve studies from all over the world which permitted a contextualised analysis of each SDH. We were able to answer to our research question which to the best of our knowledge has not been addressed in a systematic review before. However, we focused our search on peer-reviewed studies which might have left reports from grey literature or from non-indexed articles. No studies from Africa or South America were retrieved, regions with stark inequalities; this might be due to our choices of databases as African and South American studies are less likely to be indexed in the databases we chose [76,77].

The high heterogeneity of studies also warrants some discussion, especially in the components analysed which differ greatly between studies. This was due to the data available and the perspective that informed each analysis. Some studies included dental costs in their calculation of direct medical costs; some included cost of transportation as indirect costs while others considered this a direct non-medical cost. Although out-of-pocket expenditure is the dominant cost-bearing mechanism in several included countries, explicit OOP cost components were not reported. This underreporting is concerning given that OOP expenditure is the metric most directly linked to financial hardship and catastrophic health expenditure at the household level. Standardised guidelines on the reporting of cost studies might help overcome this limitation in the future and improve synthesis of the wealth of information provided. Future cost-of-illness studies in LMIC settings should disaggregate and report OOP expenditure as a distinct cost component.

Not all studies reported statistical analysis by SDH nor adjusted estimates. The lack of adjustment for confounders such as age, comorbidities, or disease severity limits the ability to draw causal conclusions about the independent effect of each SDH on costs. We have indicated throughout Figs 2 and 3, and Table 3, whether each reported difference reflects crude or adjusted analysis, allowing readers to weight findings accordingly.

Overall, this review retrieved more studies from countries with fragmented healthcare systems where healthcare is not universal and not free at the point of delivery. In countries where rural living is highly prevalent, living in rural areas is associated with decreased direct healthcare costs. In countries where healthcare coverage is not universal, being uninsured and from an ethnic minority is associated with decreased direct healthcare costs. As previously stated, this pattern might reflect healthcare foregone, determined by ability and willingness to pay rather than clinical need; however, this needs to be further evaluated. Studies should also assess how different SDHs behave together and impact costs. The gradients generated by unequal wealth and opportunity distribution are rarely unidimensional. People with less access to education are more likely to be unemployed or have low paid jobs which will lead to lower income. This vicious cycle of inequality needs to be further studied from a cost perspective to inform policies. We observe that individuals with higher incomes, better employment, and health insurance tend to incur in greater indirect costs compared to their less well-off counterparts. However, it would be necessary to know how this cost translates into total family income to have a better understanding of the true impact of the disease on the patient.

Future research should prioritise three areas. First, studies from Africa and Latin America are needed to determine whether the foregone care and accumulated burden framework applies in contexts with different healthcare financing structures and SDH gradients. Second, longitudinal studies tracking costs alongside healthcare utilisation over time are needed to test whether foregone care in early disease stages predicts accumulated burden later, which cross-sectional studies cannot capture. Third, standardised cost reporting guidelines for SDH-stratified analyses would improve comparability across studies, particularly regarding the classification of direct, indirect, and intangible costs.

This review makes several novel contributions. It is, to the best of our knowledge, the first systematic synthesis of T2DM costs by SDH. It demonstrates that the direction of cost inequality is not universal but is moderated by healthcare financing model. It proposes a conceptual framework for interpreting cost inequalities in chronic disease that warrants further empirical testing. Finally, it identifies critical geographic gaps in the literature, particularly the absence of evidence from Africa and Latin America, where SDH-driven inequalities are most pronounced.

Inequality is a social and political construct. Societies have many options for organisation, and choosing one approach over others generates divisions of wealth [78]. This review highlights the differences in the economic consequences in the treatment of T2DM by residence, insurance status, and ethnicity. Addressing these disparities is crucial to ensure healthcare delivery is based on true clinical need rather than socio-economic factors.

## Supporting information

S1 File PRISMA checklist. (DOC)

S2 File Full search strategies for all databases. (DOCX)

## References

[1] McCartney G, Bartley M, Dundas R, Katikireddi SV, Mitchell R, Popham F, et al. Theorising social class and its application to the study of health inequalities. SSM Popul Health. 2018;7:015–15. doi: 10.1016/j.ssmph.2018.10.015 31297431 PMC6598164

[2] McCartney G, Dickie E, Escobar O, Collins C. Health inequalities, fundamental causes and power: towards the practice of good theory. Sociol Health Illn. 2021;43(1):20–39. doi: 10.1111/1467-9566.13181 33222244 PMC7894306

[3] Marmot M. Social determinants of health inequalities. Lancet. 2005;365(9464):1099–104. doi: 10.1016/S0140-6736(05)71146-6 15781105

[4] World Health Organization WHO. Operational framework for monitoring social determinants of health equity. Geneva: World Health Organization. 2024.

[5] Allen L, Williams J, Townsend N, Mikkelsen B, Roberts N, Foster C, et al. Socioeconomic status and non-communicable disease behavioural risk factors in low-income and lower-middle-income countries: a systematic review. Lancet Glob Health. 2017;5(3):e277–89. doi: 10.1016/S2214-109X(17)30058-X 28193397 PMC5673683

[6] Cockerham WC, Hamby BW, Oates GR. The Social Determinants of Chronic Disease. Am J Prev Med. 2017;52(1S1):S5–12. doi: 10.1016/j.amepre.2016.09.010 27989293 PMC5328595

[7] Tatulashvili S, Fagherazzi G, Dow C, Cohen R, Fosse S, Bihan H. Socioeconomic inequalities and type 2 diabetes complications: A systematic review. Diabetes Metab. 2020;46(2):89–99. doi: 10.1016/j.diabet.2019.11.001 31759171

[8] Walker RJ, Smalls BL, Campbell JA, Strom Williams JL, Egede LE. Impact of social determinants of health on outcomes for type 2 diabetes: a systematic review. Endocrine. 2014;47(1):29–48. doi: 10.1007/s12020-014-0195-0 24532079 PMC7029167

[9] Nagar SD, Nápoles AM, Jordan IK, Mariño-Ramírez L. Socioeconomic deprivation and genetic ancestry interact to modify type 2 diabetes ethnic disparities in the United Kingdom. EClinicalMedicine. 2021;37:100960. doi: 10.1016/j.eclinm.2021.100960 34386746 PMC8343245

[10] Kilvert A, Fox C. Health inequalities and diabetes. Practical Diabetes. 2023;40(1):19. doi: 10.1002/pdi.2435

[11] Bommer C, Sagalova V, Heesemann E, Manne-Goehler J, Atun R, Bärnighausen T. Global economic burden of diabetes in adults: projections from 2015 to 2030. Diabetes Care. 2018;41:963–70. doi: 10.2337/dc17-1962 29475843

[12] Tharkar S, Devarajan A, Kumpatla S, Viswanathan V. The socioeconomics of diabetes from a developing country: a population based cost of illness study. Diabetes Res Clin Pract. 2010;89(3):334–40. doi: 10.1016/j.diabres.2010.05.009 20538363

[13] Asaria M, Doran T, Cookson R. The costs of inequality: whole-population modelling study of lifetime inpatient hospital costs in the English National Health Service by level of neighbourhood deprivation. J Epidemiol Community Health. 2016;70(10):990–6. doi: 10.1136/jech-2016-207447 27189975 PMC5036206

[14] Hanratty B, Burström B, Walander A, Whitehead M. Inequality in the face of death? Public expenditure on health care for different socioeconomic groups in the last year of life. J Health Serv Res Policy. 2007;12(2):90–4. doi: 10.1258/135581907780279585 17407658

[15] Page MJ, McKenzie JE, Bossuyt PM, Boutron I, Hoffmann TC, Mulrow CD, et al. The PRISMA 2020 statement: an updated guideline for reporting systematic reviews. BMJ. 2021;:n71. doi: 10.1136/bmj.n71 PMC8005924 33782057

[16] Islam MM. The gradient of social determinants of health and related inequalities and early childhood development: Analysis of two rounds of a cross-sectional survey. J Paediatr Child Health. 2024;60(11):716–23. doi: 10.1111/jpc.16667 39264037

[17] De Paoli M, Zakharia A, Werstuck GH. The Role of Estrogen in Insulin Resistance. The American Journal of Pathology. 2021;191(9):1490–8. doi: 10.1016/j.ajpath.2021.05.011 34102108

[18] Yan H, Yang W, Zhou F, Li X, Pan Q, Shen Z, et al. Estrogen Improves Insulin Sensitivity and Suppresses Gluconeogenesis via the Transcription Factor Foxo1. Diabetes. 2019;68(2):291–304. doi: 10.2337/db18-0638 30487265 PMC6341301

[19] Ellison GTH, Smart A, Tutton R, Outram SM, Ashcroft R, Martin P. Racial categories in medicine: a failure of evidence-based practice?. PLoS Med. 2007;4:e287. doi: 10.1371/journal.pmed.0040287 PMC1989752 17896860

[20] Lewis C, Cohen PR, Bahl D, Levine EM, Khaliq W. Race and Ethnic Categories: A Brief Review of Global Terms and Nomenclature. Cureus. 2023;15:e41253. doi: 10.7759/cureus.41253 PMC10389293 37529803

[21] Larg A, Moss JR. Cost-of-Illness Studies. PharmacoEconomics. 2011;29(8):653–71. doi: 10.2165/11588380-000000000-00000 21604822

[22] McGuiness LA. Introduction to robvis, a visualization tool for risk-of-bias assessments. 2019. https://cran.r-project.org/web/packages/robvis/vignettes/Introduction_to_robvis.html

[23] Afroz A, Alam K, Ali L, Karim A, Alramadan MJ, Habib SH, et al. Type 2 diabetes mellitus in Bangladesh: a prevalence based cost-of-illness study. BMC Health Serv Res. 2019;19(1):601. doi: 10.1186/s12913-019-4440-3 31455307 PMC6712789

[24] Le C, Lin L, Jun D, Jianhui H, Keying Z, Wenlong C, et al. The economic burden of type 2 diabetes mellitus in rural southwest China. Int J Cardiol. 2013;165(2):273–7. doi: 10.1016/j.ijcard.2011.08.039 21908062

[25] Lee JA, Liu CF, Sales AE. Racial and ethnic differences in diabetes care and health care use and costs. Preventing Chronic Disease. 2006;3:A85. PMC1636720 16776886

[26] Li R, Bilik D, Brown MB, Zhang P, Ettner SL, Ackermann RT, et al. Medical costs associated with type 2 diabetes complications and comorbidities. Am J Manag Care. 2013;19(5):421–30. 23781894 PMC4337403

[27] Daniel A, Sangeetha S, Madonna JD. Health expenditure patterns among rural and urban people with diabetes mellitus- a prospective, comparative study. Journal of Cardiovascular Disease Research. 2021;12.

[28] Dawson AZ, Bishu KG, Walker RJ, Egede LE. Trends in Medical Expenditures by Race/Ethnicity in Adults with Type 2 Diabetes 2002-2011. J Natl Med Assoc. 2021;113(1):59–68. doi: 10.1016/j.jnma.2020.07.008 32773238 PMC7865019

[29] D E, Endarti D, Widayanti AW, Rahmawati EA. Khaher N. Cost of illness of type 2 diabetes mellitus from the patient’s perspective: A study from several primary healthcare centres and a hospital in Yogyakarta, Indonesia. International Journal of Research in Pharmaceutical Sciences. 2020;11:7442–53. doi: 10.26452/IJRPS.V11I4.3934

[30] Guo J, Wu Y, Deng X, Liu Z, Chen L, Huang Y. Association between social determinants of health and direct economic burden on middle-aged and elderly individuals living with diabetes in China. PLoS One. 2021;16(4):e0250200. doi: 10.1371/journal.pone.0250200 33857252 PMC8049277

[31] Hazel-Fernandez L, Li Y, Nero D, Moretz C, Slabaugh L, Meah Y, et al. Racial/ethnic and gender differences in severity of diabetes-related complications, health care resource use, and costs in a Medicare population. Popul Health Manag. 2015;18(2):115–22. doi: 10.1089/pop.2014.0038 25290044

[32] Hu R, Shi L, Pierre G, Zhu J, Lee D-C. Diabetes and medical expenditures among non-institutionalized U.S. adults. Diabetes Res Clin Pract. 2015;108(2):223–34. doi: 10.1016/j.diabres.2015.02.016 25771306

[33] Jacobs P, Blanchard JF, James RC, Depew N. Excess costs of diabetes in the Aboriginal population of Manitoba, Canada. Can J Public Health. 2000;91(4):298–301. doi: 10.1007/BF03404293 10986790 PMC6980072

[34] Jing Z, Chu J, Imam Syeda Z, Zhang X, Xu Q, Sun L, et al. Catastrophic health expenditure among type 2 diabetes mellitus patients: A province-wide study in Shandong, China. J Diabetes Investig. 2019;10(2):283–9. doi: 10.1111/jdi.12901 30044060 PMC6400173

[35] Kim HJ, Chun KH, Kim DJ, Han SJ, Kim YS, Woo JT, et al. Utilization patterns and cost of complementary and alternative medicine compared to conventional medicine in patients with type 2 diabetes mellitus. Diabetes Res Clin Pract. 2011;93(1):115–22. doi: 10.1016/j.diabres.2011.03.031 21524810

[36] König H, Rommel A, Baumert J, Schmidt C, König H-H, Brettschneider C, et al. Excess costs of type 2 diabetes and their sociodemographic and clinical determinants: a cross-sectional study using data from the German Health Interview and Examination Survey for Adults (DEGS1). BMJ Open. 2021;11(4):e043944. doi: 10.1136/bmjopen-2020-043944 33883150 PMC8061816

[37] Kumar A, Nagpal J, Bhartia A. Direct cost of ambulatory care of type 2 diabetes in the middle and high income group populace of Delhi: the DEDICOM survey. J Assoc Physicians India. 2008;56:667–74. 19086352

[38] Khowaja LA, Khuwaja AK, Cosgrove P. Cost of diabetes care in out-patient clinics of Karachi, Pakistan. BMC Health Serv Res. 2007;7:189. doi: 10.1186/1472-6963-7-189 18028552 PMC2206019

[39] Simmons M, Bishu KG, Williams JS, Walker RJ, Dawson AZ, Egede LE. Racial and Ethnic Differences in Out-of-Pocket Expenses among Adults with Diabetes. J Natl Med Assoc. 2019;111(1):28–36. doi: 10.1016/j.jnma.2018.04.004 30129486 PMC7995684

[40] Sun X, Liabsuetrakul T, Xie X, Liu P, Zhang Y, Wang Z. Ethnic Disparity in Annual Healthcare Expenditures for Type 2 Diabetes Mellitus in Ningxia, China. J Racial Ethn Health Disparities. 2018;5(6):1381–8. doi: 10.1007/s40615-018-0488-8 29600352

[41] Williams JS, Lu K, Akinboboye O, Olukotun O, Zhou Z, Nagavally S. Trends in Obesity and Medical Expenditure among Women with Diabetes, 2008-2016: Differences by Race/Ethnicity. Ethnicity & Disease. 2020;30:621–8. doi: 10.18865/ed.30.4.621 32989362 PMC7518529

[42] Rice T, Rosenau P, Unruh LY, Barnes AJ, Saltman RB, van Ginneken E. United States of America: health system review. Health Syst Transit. 2013;15(3):1–431. 24025796

[43] Census Bureau US. Health Insurance Coverage in the United States: 2020. 2020. https://www.census.gov/library/publications/2021/demo/p60-274.html

[44] Centers for Medicare & Medicaid Services. NHE Fact Sheet. 2024. https://www.cms.gov/data-research/statistics-trends-and-reports/national-health-expenditure-data/nhe-fact-sheet#

[45] Fu W, Zhao S, Zhang Y, Chai P, Goss J. Research in health policy making in China: out-of-pocket payments in Healthy China 2030. BMJ. 2018. doi: 10.1136/bmj.k234 PMC5797981 29437565

[46] Yi B. An overview of the Chinese healthcare system. Hepatobiliary Surg Nutr. 2021;10(1):93–5. doi: 10.21037/hbsn-2021-3 33575292 PMC7867737

[47] Huq NM, Al-Amin AQ, Howlader SR, Kabir MA. Paying out of pocket for healthcare in Bangladesh - a burden on poor?. Iranian Journal of Public Health. 2015;44:1024–5. 26576387 PMC4645756

[48] Sriram S, Albadrani M. Impoverishing effects of out-of-pocket healthcare expenditures in India. J Family Med Prim Care. 2022;11(11):7120–8. doi: 10.4103/jfmpc.jfmpc_590_22 36993034 PMC10041239

[49] Naz L, Sriram S. Out-of-pocket expenditures associated with double disease burden in Pakistan: a quantile regression analysis. BMC Public Health. 2024;24(1):801. doi: 10.1186/s12889-024-18320-4 38486277 PMC10938732

[50] Asante A, Cheng Q, Susilo D, Satrya A, Haemmerli M, Fattah RA, et al. The benefits and burden of health financing in Indonesia: analyses of nationally representative cross-sectional data. Lancet Glob Health. 2023;11(5):e770–80. doi: 10.1016/S2214-109X(23)00064-5 37061314

[51] Martin D, Miller AP, Quesnel-Vallée A, Caron NR, Vissandjée B, Marchildon GP. Canada’s universal health-care system: achieving its potential. Lancet. 2018;391(10131):1718–35. doi: 10.1016/S0140-6736(18)30181-8 29483027 PMC7138369

[52] Lee SY, Chun CB, Lee YG, Seo NK. The national health insurance system as one type of new typology: The case of South Korea and Taiwan. Health Policy. 2008;85:105–13. doi: 10.1016/j.healthpol.2007.07.006 17709152

[53] Park E, Choi S. Who Benefits from the Fixed Copayment of Medical and Pharmaceutical Expenditure among the Korean Elderly?. Int J Environ Res Public Health. 2020;17(21):8118. doi: 10.3390/ijerph17218118 33153173 PMC7663709

[54] Döring A, Paul F. The German healthcare system. EPMA J. 2010;1(4):535–47. doi: 10.1007/s13167-010-0060-z 23199108 PMC3405354

[55] Greß S. Private health insurance in Germany: consequences of a dual system. Healthc Policy. 2007;3(2):29–37. doi: 10.12927/hcpol.2007.19389 19305777 PMC2645182

[56] Shin S. Ability to pay and catastrophic health expenditure of urban and rural deceased households over the past decade (2009-2018). Rural and Remote Health. 2024. doi: 10.22605/RRH8566 38772696

[57] Guo B, Xie X, Wu Q, Zhang X, Cheng H, Tao S, et al. Inequality in the health services utilization in rural and urban china. Medicine. 2020;99(2):e18625. doi: 10.1097/md.0000000000018625 PMC6959938 31914043

[58] Zhao Y, Tang S, Mao W, Akinyemiju T. Socio-Economic and Rural-Urban Differences in Healthcare and Catastrophic Health Expenditure Among Cancer Patients in China: Analysis of the China Health and Retirement Longitudinal Study. Frontiers in Public Health. 2022;9. doi: 10.3389/fpubh.2021.779285 PMC8787105 35087783

[59] Chawla NS. Unveiling the ABCs: Identifying India’s Healthcare Service Gaps. Cureus. 2023;15(7):e42398. doi: 10.7759/cureus.42398 37621782 PMC10446776

[60] Mazid MA, Azam MG. Health Equity in Bangladesh: A Comparative Review and Recommendations for Policy and Practice. SSRN Journal. 2024. doi: 10.2139/ssrn.4726520

[61] Basu J. Research on Disparities in Primary Health Care in Rural versus Urban Areas: Select Perspectives. Int J Environ Res Public Health. 2022;19(12):7110. doi: 10.3390/ijerph19127110 35742359 PMC9222532

[62] National Academies of Sciences, Engineering, and Medicine. Health-care utilization as a proxy in disability determination. Washington D.C. 2020. 29782136

[63] Jansen T, Rademakers J, Waverijn G, Verheij R, Osborne R, Heijmans M. The role of health literacy in explaining the association between educational attainment and the use of out-of-hours primary care services in chronically ill people: a survey study. BMC Health Serv Res. 2018;18(1):394. doi: 10.1186/s12913-018-3197-4 29855365 PMC5984471

[64] Schumacher JR, Hall AG, Davis TC, Arnold CL, Bennett RD, Wolf MS. Potentially Preventable Use of Emergency Services. Medical Care. 2013;51:654–8. doi: 10.1097/MLR.0b013e3182992c5a 23703649 PMC3756810

[65] Barlow P, Mohan G, Nolan A, Lyons S. Area-level deprivation and geographic factors influencing utilisation of General Practitioner services. SSM Popul Health. 2021;15:100870. doi: 10.1016/j.ssmph.2021.100870 34386571 PMC8342788

[66] van der Beek AJ, Kunst AE. How can we break the vicious circle between poor health and exit from paid employment?. Scand J Work Environ Health. 2019;45(4):321–3. doi: 10.5271/sjweh.3838 31187870

[67] Yassin AS, Beckles GL, Messonnier ML. Disability and its economic impact among adults with diabetes. J Occup Environ Med. 2002;44(2):136–42. doi: 10.1097/00043764-200202000-00008 11851214

[68] Manodpitipong A, Saetung S, Nimitphong H, Siwasaranond N, Wongphan T, Sornsiriwong C, et al. Night-shift work is associated with poorer glycaemic control in patients with type 2 diabetes. J Sleep Res. 2017;26(6):764–72. doi: 10.1111/jsr.12554 28548389

[69] Carlsson S, Andersson T, Talbäck M, Feychting M. Incidence and prevalence of type 2 diabetes by occupation: results from all Swedish employees. Diabetologia. 2020;63(1):95–103. doi: 10.1007/s00125-019-04997-5 31570970 PMC6890587

[70] Mathur R, Farmer RE, Eastwood SV, Chaturvedi N, Douglas I, Smeeth L. Ethnic disparities in initiation and intensification of diabetes treatment in adults with type 2 diabetes in the UK, 1990-2017: A cohort study. PLoS Med. 2020;17(5):e1003106. doi: 10.1371/journal.pmed.1003106 32413037 PMC7228040

[71] Chudasama YV, Zaccardi F, Coles B, Gillies CL, Hvid C, Seidu S, et al. Ethnic, social and multimorbidity disparities in therapeutic inertia: A UK primary care observational study in patients newly diagnosed with type 2 diabetes. Diabetes Obes Metab. 2021;23(11):2437–45. doi: 10.1111/dom.14482 34189827

[72] Su Y, Lu Y, Li W, Xue M, Chen C, Haireti M, et al. Prevalence and Correlation of Metabolic Syndrome: A Cross-Sectional Study of Nearly 10 Million Multi-Ethnic Chinese Adults. Diabetes Metab Syndr Obes. 2020;13:4869–83. doi: 10.2147/DMSO.S278346 33335411 PMC7737555

[73] Wu J, Cheng X, Qiu L, Xu T, Zhu G, Han J, et al. Prevalence and Clustering of Major Cardiovascular Risk Factors in China. Medicine. 2016;95(10):e2712. doi: 10.1097/md.0000000000002712 PMC4998852 26962771

[74] Cheran K, Murthy C, Bornemann EA, Kamma HK, Alabbas M, Elashahab M, et al. The Growing Epidemic of Diabetes Among the Indigenous Population of Canada: A Systematic Review. Cureus. 2023;15(3):e36173. doi: 10.7759/cureus.36173 37065334 PMC10103803

[75] Ananian S, Dellaferrera G. Employment and wage disparities between rural and urban areas. 2022.

[76] Asubiaro TV, Onaolapo S. A comparative study of the coverage of African journals in Web of Science, Scopus, and CrossRef. Asso for Info Science & Tech. 2023;74(7):745–58. doi: 10.1002/asi.24758

[77] Asubiaro T, Onaolapo S, Mills D. Regional disparities in Web of Science and Scopus journal coverage. Scientometrics. 2024;129(3):1469–91. doi: 10.1007/s11192-024-04948-x

[78] Piketty T. A brief history of equality. The Belknap Press of Harvard University Press. 2021.

