# Supplementary material for: The economic burden of Type 2 Diabetes by social determinants of health: A systematic review
Source: PLoS One. 2026 Jul 27;21(7):e0354198. doi: 10.1371/journal.pone.0354198 (PMC13405126; doi:10.1371/journal.pone.0354198)
Supplement: S2 File — (DOCX) [file pone.0354198.s002.docx]

**Supplementary material**

**Supplementary material 1. Search strategy**

| **MEDLINE** | | |
| --- | --- | --- |
| **N°** | **Search command** | **Hits** |
| **1** | (((type 2 diabetes[MeSH Major Topic]) OR (type 2 diabetes mellitus[Title/Abstract])) OR (non insulin dependent diabetes[Title/Abstract])) OR (NIDDM[Text Word] OR T2DM[Text Word] OR T2D[Text Word]) | 177,207 |
| **2** | "diabetes mellitus, type 1"[MeSH Major Topic] OR "diabetes mellitus, type 1"[MeSH Terms] OR "diabetes insipidus"[MeSH Terms] OR "diabetes, gestational"[MeSH Terms] OR "IDDM"[Text Word] OR "T1D"[Text Word] OR "T1DM"[Text Word] | 115,320 |
| **3** | #1 NOT #2 | 161,068 |
| **4** | "cost of illness"[MeSH Major Topic] OR "illness cost"[Title/Abstract] OR "sickness cost"[Title/Abstract] OR "burden of illness"[Title/Abstract] OR "disease cost"[Title/Abstract] OR "disease costs"[Title/Abstract] OR "health care cost"[Title/Abstract] OR "hospital cost"[Title/Abstract] OR "indirect cost"[Title/Abstract] OR "productivity costs"[Title/Abstract] OR "productivity lost"[Title/Abstract] OR "productivity loss"[Title/Abstract] OR "absenteeism cost"[Title/Abstract] OR "human capital"[Title/Abstract] OR "economic burden"[Title/Abstract] OR "cost of illness"[MeSH Terms] | 41,452 |
| **5** | "social determinants of health"[MeSH Major Topic] OR "socioeconomic factor"[Title/Abstract] OR ("economic"[Title/Abstract] AND "social factors"[Title/Abstract]) OR "social inequality"[Title/Abstract] OR "standard of living"[Title/Abstract] OR "low income"[Title/Abstract] OR "ethnic group"[Title/Abstract] OR "healthcare disparities"[MeSH Terms] | 86,422 |
| **6** | #4 AND #5 | 57,651 |
| **7** | diabet* OR #1 | 944,812 |
| **8** | #7 NOT #2 | 831,079 |
| **9** | #8 AND #4 AND #5 | 2,976 |
| **10** | #9 restricted to Humans | 2,494 |
| **11** | ("systematic review"[Publication Type] OR "REVIEW"[Publication Type] OR "meta-analysis"[Publication Type] OR "systematic review"[Title]) AND (humans[Filter]) | 2,655,758 |
| **12** | #10 NOT #11 | 1844 |
| **Search string for EconLit and other EMBASE based databases** | | |
| mainsubject(diabetes type 2) OR summary(T2DM) OR summary(type 2 diabetes mellitus) NOT mainsubject(type 1 diabetes) NOT mainsubject(gestational diabetes) NOT mainsubject(diabetes insipidus) AND (absenteeism OR COST OF DISEASE OR cost of illness OR burden of disease OR PRODUCTIVITY) AND (SOCIAL DETERMINANTS ) | | |
