# Supplementary material for: The economic burden of Type 2 Diabetes by social determinants of health: A systematic review
Source: PLoS One. 2026 Jul 27;21(7):e0354198. doi: 10.1371/journal.pone.0354198 (PMC13405126; doi:10.1371/journal.pone.0354198)
Supplement: Attachment — Submitted filename: Dear Editor.docx [file pone.0354198.s003.docx]

***Dear Editor***

First off, the authors did a great job setting the stage. The introduction clearly lays out why this is such an important question—type 2 diabetes is a massive global problem, and we know social factors play a huge role in who gets it and how they fare. But this review smartly focuses on a less-discussed angle: the actual financial cost of those inequalities. Their methods are top-notch too; they registered the study on PROSPERO, followed PRISMA guidelines for transparency, and searched a wide range of databases, which gives me a lot of confidence that they found the relevant research out there. The way they've presented all the data in the tables and figures is also really clear and helps you see the patterns at a glance.

Now, where things get a bit tricky, and this is a common challenge in this type of research, is the sheer variety of how different studies define and measure things like 'education' or 'income.' This makes it hard to compare apples to apples, which is why they couldn't do a statistical meta-analysis and had to describe the results narratively. It's not the authors' fault at all; it's just the state of the literature, but it's a significant limitation that means we have to interpret the findings with a bit of caution. I also noticed they pulled a lot of data from the US and China, which is great, but it makes me wonder how these findings apply to places in Africa or South America, which are barely represented. The story of cost inequality might be very different there.

And while they are clearly experts at analyzing the numbers, I felt the discussion could have gone a step further to really hammer home the 'so what?' for policymakers. For example, they highlight that in countries where people pay for care out-of-pocket, the poor and rural populations often spend less on treatment, likely because they simply can't afford it and may delay care. But in countries with universal healthcare, the pattern flips, and sometimes the less educated or those facing other disadvantages end up costing the system more, perhaps because of lower health literacy leading to more expensive emergency room visits down the line. That's a crucial insight, and the paper could have ended by more forcefully suggesting what this means for real-world policy—like the need for better health education programs or subsidized transportation for rural patients.

But all in all, this is a high-quality review. It's rigorous, it's relevant, and it shines a light on a really important issue that doesn't get enough attention. My final judgment is that this is a valuable and well-executed study that adds a lot to our understanding of the economics of diabetes. It's a professional, expert piece of work, no doubt about it, and any researcher or policymaker in this field would benefit from reading it.

1. **Title and Abstract**

- Could explicitly state the *primary outcome* (e.g., "cost differences by SDH") in the abstract.
- "Cost of inequalities" is slightly ambiguous; "economic burden of health disparities" might be clearer.

**2. Introduction**

- Overly broad definition of SDH (e.g., includes insurance, which is more of a *healthcare access* factor).
- Could better distinguish *biological sex* from *gender* to justify exclusion.

**3. Methods**

- **SDH Operationalization:** Inconsistent definitions (e.g., "ethnicity" vs. "race").
- **Risk of Bias:** No assessment for publication bias (e.g., funnel plots).
- **Data Synthesis:** Percentage differences are useful but limit comparability due to heterogeneous SDH categorizations.

**4. Results**

- **Heterogeneity:** High variability in cost components (e.g., "indirect costs" defined inconsistently) impedes synthesis.
- **Statistical Reporting:** Some studies lack adjusted p-values (e.g., Figure 2), weakening causal inference.
- **OOP Costs:** Underreported despite relevance in low/middle-income countries (LMICs).

**5. Discussion**

- **Policy Implications:** Underdeveloped. Could suggest actionable strategies (e.g., insurance expansions, rural infrastructure).
- **Generalizability:** Overreliance on US/China data (13/22 studies) limits global applicability.
- **Gender Exclusion:** Justification is weak; gender influences health-seeking behavior and costs.

**6. Conclusion**

- Lacks forward-looking recommendations (e.g., "future studies should...").
- Does not reiterate the public health significance of addressing cost inequalities.

**7. Overall Assessment**

- **Heterogeneity:** Limits quantitative synthesis; meta-analysis was infeasible.
- **Geographic Bias:** Underrepresentation of Africa/Latin America.
- **SDH Scope:** Some factors (e.g., insurance) may not align with WHO SDH framework.

**8. Recommendations for Improvement**

1. **Clarify SDH Definitions:** Align with WHO SDH framework (e.g., exclude insurance).
2. **Enhance Statistical Synthesis:** Use random-effects models where possible; report confidence intervals.
3. **Expand Policy Discussion:** Propose interventions (e.g., subsidized care for low-income groups).
4. **Address Gaps:** Include gender, LMICs beyond Asia, and standardized cost components.

**9. Final Judgment**

This is a high-quality systematic review with methodological rigor and significant contributions to understanding diabetes cost inequalities. minor Revision to address critiques would strengthen its impact.
