# Supplementary material for: The economic burden of Type 2 Diabetes by social determinants of health: A systematic review
Source: PLoS One. 2026 Jul 27;21(7):e0354198. doi: 10.1371/journal.pone.0354198 (PMC13405126; doi:10.1371/journal.pone.0354198)
Supplement: Peer review history

# Peer review: Decision Letter 0

**DOI:** 10.1371/journal.pone.0354198.r001

**Authors:** Pasyodun Koralage Buddhika Mahesh

9 Apr 2026

PONE-D-25-68922

The cost of inequality in the treatment of Type 2 Diabetes: A systematic review

PLOS One

Dear Dr. Alarcón-Yaquetto,

Thank you for submitting your manuscript to PLOS ONE. After careful consideration, we feel that it has merit but does not fully meet PLOS ONE’s publication criteria as it currently stands. Therefore, we invite you to submit a revised version of the manuscript that addresses the points raised during the review process.

Please submit your revised manuscript by May 24 2026 11:59PM. If you will need more time than this to complete your revisions, please reply to this message or contact the journal office at plosone@plos.org. When you're ready to submit your revision, log on to https://www.editorialmanager.com/pone/ and select the 'Submissions Needing Revision' folder to locate your manuscript file.

Please include the following items when submitting your revised manuscript:

- A letter that responds to each point raised by the academic editor and reviewer(s). You should upload this letter as a separate file labeled 'Response to Reviewers'.
- A marked-up copy of your manuscript that highlights changes made to the original version. You should upload this as a separate file labeled 'Revised Manuscript with Track Changes'.
- An unmarked version of your revised paper without tracked changes. You should upload this as a separate file labeled 'Manuscript'.

If you would like to make changes to your financial disclosure, please include your updated statement in your cover letter. Guidelines for resubmitting your figure files are available below the reviewer comments at the end of this letter.

If applicable, we recommend that you deposit your laboratory protocols in protocols.io to enhance the reproducibility of your results. Protocols.io assigns your protocol its own identifier (DOI) so that it can be cited independently in the future. For instructions see: https://journals.plos.org/plosone/s/submission-guidelines#loc-laboratory-protocols. Additionally, PLOS ONE offers an option for publishing peer-reviewed Lab Protocol articles, which describe protocols hosted on protocols.io. Read more information on sharing protocols at https://plos.org/protocols?utm_medium=editorial-email&utm_source=authorletters&utm_campaign=protocols.

As the corresponding author, your ORCID iD is verified in the submission system and will appear in the published article. PLOS supports the use of ORCID, and we encourage all coauthors to register for an ORCID iD and use it as well. Please encourage your coauthors to verify their ORCID iD within the submission system before final acceptance, as unverified ORCID iDs will not appear in the published article. *Only*  the individual author can complete the verification step; PLOS staff *cannot*  verify ORCID iDs on behalf of authors.

We look forward to receiving your revised manuscript.

Kind regards,

Pasyodun Koralage Buddhika Mahesh

Academic Editor

PLOS One

Journal Requirements:

When submitting your revision, we need you to address these additional requirements.

1. Please ensure that your manuscript meets PLOS ONE's style requirements, including those for file naming. The PLOS ONE style templates can be found at

https://journals.plos.org/plosone/s/file?id=wjVg/PLOSOne_formatting_sample_main_body.pdf and https://journals.plos.org/plosone/s/file?id=ba62/PLOSOne_formatting_sample_title_authors_affiliations.pdf

2. Thank you for stating the following financial disclosure:

[This study was funded by the Economic and Social Research Council, London Indisciplinary Social Science Doctoral Training Partnership (LISS-DTP ES/P000703/1) Project Reference 2606816].

Please state what role the funders took in the study.  If the funders had no role, please state: "The funders had no role in study design, data collection and analysis, decision to publish, or preparation of the manuscript."

If this statement is not correct you must amend it as needed.

Please include this amended Role of Funder statement in your cover letter; we will change the online submission form on your behalf.

3. Please remove any funding-related text from the manuscript. Funding information should not appear in any section or other areas of your manuscript. We will only publish funding information present in the Funding Statement section of the online submission form.

4. Please include captions for your Supporting Information files at the end of your manuscript, and update any in-text citations to match accordingly. Please see our Supporting Information guidelines for more information: http://journals.plos.org/plosone/s/supporting-information.

If the reviewer comments include a recommendation to cite specific previously published works, please review and evaluate these publications to determine whether they are relevant and should be cited. There is no requirement to cite these works unless the editor has indicated otherwise.

[Note: HTML markup is below. Please do not edit.]

Reviewers' comments:

Reviewer's Responses to Questions

**Comments to the Author**

1. Is the manuscript technically sound, and do the data support the conclusions?

The manuscript must describe a technically sound piece of scientific research with data that supports the conclusions. Experiments must have been conducted rigorously, with appropriate controls, replication, and sample sizes. The conclusions must be drawn appropriately based on the data presented.

Reviewer #1: Partly

Reviewer #2: Yes

Reviewer #3: Partly

Reviewer #4: Partly

Reviewer #5: Yes

**********

2. Has the statistical analysis been performed appropriately and rigorously?

Reviewer #1: N/A

Reviewer #2: Yes

Reviewer #3: Yes

Reviewer #4: N/A

Reviewer #5: N/A

**********

3. Have the authors made all data underlying the findings in their manuscript fully available?

The PLOS Data policy requires authors to make all data underlying the findings described in their manuscript fully available without restriction, with rare exception (please refer to the Data Availability Statement in the manuscript PDF file). The data should be provided as part of the manuscript or its supporting information, or deposited to a public repository. For example, in addition to summary statistics, the data points behind means, medians and variance measures should be available. If there are restrictions on publicly sharing data—e.g. participant privacy or use of data from a third party—those must be specified.

Reviewer #1: No

Reviewer #2: Yes

Reviewer #3: Yes

Reviewer #4: No

Reviewer #5: Yes

**********

4. Is the manuscript presented in an intelligible fashion and written in standard English?

PLOS ONE does not copyedit accepted manuscripts, so the language in submitted articles must be clear, correct, and unambiguous. Any typographical or grammatical errors should be corrected at revision, so please note any specific errors here.

Reviewer #1: Yes

Reviewer #2: Yes

Reviewer #3: Yes

Reviewer #4: Yes

Reviewer #5: Yes

**********

5. Review Comments to the Author

Please use the space provided to explain your answers to the questions above. You may also include additional comments for the author, including concerns about dual publication, research ethics, or publication ethics. (Please upload your review as an attachment if it exceeds 20,000 characters)

Reviewer #1: Methodology (Systematic Review Standards)

Although the article is presented as a systematic review, the details of the search strategy, inclusion/exclusion criteria, and study selection process are not sufficiently clear to allow for full replication of the study.

Minimum information about the Quality and Risk of Bias Assessment

No formal assessment of methodological quality or risk of bias of included studies was reported, which limits the validity and weight of the results. They only presented a table about the risk of bias without any explanation about the method.

Descriptive Rather Than Analytical Synthesis

The findings are largely descriptive, and no comparative or framework analysis is provided to explain how inequalities affect treatment costs.

Limited Novelty and Added Value

The novelty of the study compared to previous reviews is not clearly stated, and many of the results are replications of well-known findings in the existing literature.

Policy and Practical Implications

The policy and practical implications are stated in general terms and do not lead to specific, actionable

Reviewer #2: Dear Editor

First off, the authors did a great job setting the stage. The introduction clearly lays out why this is such an important question—type 2 diabetes is a massive global problem, and we know social factors play a huge role in who gets it and how they fare. But this review smartly focuses on a less-discussed angle: the actual financial cost of those inequalities. Their methods are top-notch too; they registered the study on PROSPERO, followed PRISMA guidelines for transparency, and searched a wide range of databases, which gives me a lot of confidence that they found the relevant research out there. The way they've presented all the data in the tables and figures is also really clear and helps you see the patterns at a glance.

Now, where things get a bit tricky, and this is a common challenge in this type of research, is the sheer variety of how different studies define and measure things like 'education' or 'income.' This makes it hard to compare apples to apples, which is why they couldn't do a statistical meta-analysis and had to describe the results narratively. It's not the authors' fault at all; it's just the state of the literature, but it's a significant limitation that means we have to interpret the findings with a bit of caution. I also noticed they pulled a lot of data from the US and China, which is great, but it makes me wonder how these findings apply to places in Africa or South America, which are barely represented. The story of cost inequality might be very different there.

And while they are clearly experts at analyzing the numbers, I felt the discussion could have gone a step further to really hammer home the 'so what?' for policymakers. For example, they highlight that in countries where people pay for care out-of-pocket, the poor and rural populations often spend less on treatment, likely because they simply can't afford it and may delay care. But in countries with universal healthcare, the pattern flips, and sometimes the less educated or those facing other disadvantages end up costing the system more, perhaps because of lower health literacy leading to more expensive emergency room visits down the line. That's a crucial insight, and the paper could have ended by more forcefully suggesting what this means for real-world policy—like the need for better health education programs or subsidized transportation for rural patients.

But all in all, this is a high-quality review. It's rigorous, it's relevant, and it shines a light on a really important issue that doesn't get enough attention. My final judgment is that this is a valuable and well-executed study that adds a lot to our understanding of the economics of diabetes. It's a professional, expert piece of work, no doubt about it, and any researcher or policymaker in this field would benefit from reading it.

1. Title and Abstract

• Could explicitly state the primary outcome (e.g., "cost differences by SDH") in the abstract.

• "Cost of inequalities" is slightly ambiguous; "economic burden of health disparities" might be clearer.

2. Introduction

• Overly broad definition of SDH (e.g., includes insurance, which is more of a healthcare access factor).

• Could better distinguish biological sex from gender to justify exclusion.

3. Methods

• SDH Operationalization: Inconsistent definitions (e.g., "ethnicity" vs. "race").

• Risk of Bias: No assessment for publication bias (e.g., funnel plots).

• Data Synthesis: Percentage differences are useful but limit comparability due to heterogeneous SDH categorizations.

4. Results

• Heterogeneity: High variability in cost components (e.g., "indirect costs" defined inconsistently) impedes synthesis.

• Statistical Reporting: Some studies lack adjusted p-values (e.g., Figure 2), weakening causal inference.

• OOP Costs: Underreported despite relevance in low/middle-income countries (LMICs).

5. Discussion

• Policy Implications: Underdeveloped. Could suggest actionable strategies (e.g., insurance expansions, rural infrastructure).

• Generalizability: Overreliance on US/China data (13/22 studies) limits global applicability.

• Gender Exclusion: Justification is weak; gender influences health-seeking behavior and costs.

6. Conclusion

• Lacks forward-looking recommendations (e.g., "future studies should...").

• Does not reiterate the public health significance of addressing cost inequalities.

7. Overall Assessment

• Heterogeneity: Limits quantitative synthesis; meta-analysis was infeasible.

• Geographic Bias: Underrepresentation of Africa/Latin America.

• SDH Scope: Some factors (e.g., insurance) may not align with WHO SDH framework.

8. Recommendations for Improvement

1. Clarify SDH Definitions: Align with WHO SDH framework (e.g., exclude insurance).

2. Enhance Statistical Synthesis: Use random-effects models where possible; report confidence intervals.

3. Expand Policy Discussion: Propose interventions (e.g., subsidized care for low-income groups).

4. Address Gaps: Include gender, LMICs beyond Asia, and standardized cost components.

9. Final Judgment

This is a high-quality systematic review with methodological rigor and significant contributions to understanding diabetes cost inequalities. minor Revision to address critiques would strengthen its impact.

Reviewer #3: The authors conducted a rigorous systematic review including updates from H2 of 2025. As noted in the above section, the review has been performed appropriately and rigorously, and the underlying data/references are fully transparent. The topic is of interest, and the authors did a thorough job vetting of the literature. However, the discussion and how the authors deal with the question of cost of inequality in the treatment of Type 2 Diabetes can be better fleshed out. Part of the issue is the combining of a number of disparate countries into one review. I would recommend in the discussion potentially discussing the different determinants by region - i.e. US, Asia, Europe and Latin America and come up with conclusions by region as well as by factor (i.e. income, etc). Also, I know it may be a bit beyond the scope of this article, but there could be a paragraph in the discussion re policy recommendations to mitigate these disparities. Also, I think a bit more on why T2D was selected might be important for the reader - why this condition versus something else. In summary, the authors clearly did a very thorough job of conducting this research and I believe this topic is of high import. I just think that the discussion/story telling component needs to be refined so that the reader can better ascertain how the "cost of inequality" impacts his/her region as well as globally, where there may be different policy levers depending on geography to help mitigate these disparities.

Reviewer #4: It was a systematic review with unclear question. A major revision is requested.

1- The title of the study should be updated as a clear question. I could not understand which of the following is related to your study. "Cost of inequality", "cost and inequality", "inequality in costs", or ... ?

2- In addition, you emphasized on SDH. The role of SDH should be clarified in the title.

3- The study objectives should be updated according to the above comments.

4- I suggest "thematic synthesis" to improve the quality of data synthesis and designing a thematic analysis graph.

Reviewer #5: The aim of the study is to examine the cost of treating type 2 diabetes related to psychosocial determinants of health. Overall, I do not have major issues with the methodology, except for a few minor comments, such as adding dates in the abstract or clarifying the publication period.

My major concern is with the results section. The title of the paper is confusing. After reading it, I expected to see a comparison showing the cost of type 2 diabetes based on social determinants of health. For example, I expected to see a table comparing costs across groups. However, the authors did not provide this. Instead, they categorized the findings into different social determinants of health, such as place of residence, employment, education, income, insurance, and ethnicity. This raises questions.

First, this is an international study, and comparing education levels in some countries (e.g., low‑resource countries) to countries like the United States is mismatched. My second concern relates to the findings on ethnicity and race. Except for a few developed countries, such as England and the United States, where racial categories exist, the categorization for other countries does not make sense. It seems the authors may need to revise the aim to learn the association between SDOH and T2DM treatment or expenses not in viewpoint of patients but the healthcare system or maybe both.

I have similar concerns with the discussion section. The authors selected several dimensions of social determinants of health and discussed findings, but the presentation is confusing. I am not sure how they intend to address these issues.

If I were conducting this study, I would first compare the populations in each selected article and provide more information about the study populations—for example, gender distribution, age distribution, race distribution, income distribution, and education distribution, if available. Then I would add findings from each paper, such as out‑of‑pocket payments or cost of the treatment, which might be a useful metric for comparison, or other relevant elements the authors want to highlight. After that, I would compare treatment costs.

I do not know how much the authors will benefit from my comments, but I hope they are helpful. Thank you for sharing.

Minor comments:

This is the review for PLOS One on the cost of inequality in the treatment of type 2 diabetes. The study performed a systematic review, but the timeframe is not clear. It seems the review was conducted in March 2025, going back to a study performed on February 6, 2023, with an update on October 5, 2025. However, the timeframe for the articles included in the study is not clearly stated. In some places, it is mentioned as 2000 to 2021. Please clarify the study period.

**********

6. PLOS authors have the option to publish the peer review history of their article (what does this mean?). If published, this will include your full peer review and any attached files.

If you choose “no”, your identity will remain anonymous but your review may still be made public.

**Do you want your identity to be public for this peer review?**  For information about this choice, including consent withdrawal, please see our Privacy Policy.

Reviewer #1: No

Reviewer #2: No

Reviewer #3: No

Reviewer #4: **Yes:**  Seyyed Amir Yasin Ahmadi

Reviewer #5: No

**********

[NOTE: If reviewer comments were submitted as an attachment file, they will be attached to this email and accessible via the submission site. Please log into your account, locate the manuscript record, and check for the action link "View Attachments". If this link does not appear, there are no attachment files.]

To ensure your figures meet our technical requirements, please review our figure guidelines: https://journals.plos.org/plosone/s/figures

You may also use PLOS’s free figure tool, NAAS, to help you prepare publication quality figures: https://journals.plos.org/plosone/s/figures#loc-tools-for-figure-preparation.

NAAS will assess whether your figures meet our technical requirements by comparing each figure against our figure specifications.

Attachment Submitted filename: Dear Editor.docx

# Author comment: Author response to Decision Letter 1

**DOI:** 10.1371/journal.pone.0354198.r002

13 Jun 2026

Reviewer #1: Methodology (Systematic Review Standards)

1.1 Although the article is presented as a systematic review, the details of the search strategy, inclusion/exclusion criteria, and study selection process are not sufficiently clear to allow for full replication of the study.

The review protocol was registered on PROSPERO, and the complete search strategy, including database specific search strings, is provided in Supplementary File 1. To improve reproducibility, we have expanded the Methods section to provide additional detail on the study selection and data extraction procedures. We have also clarified the dates of the original and updated searches and the databases searched.

1.2. Minimum information about the Quality and Risk of Bias Assessment

No formal assessment of methodological quality or risk of bias of included studies was reported, which limits the validity and weight of the results. They only presented a table about the risk of bias without any explanation about the method.

Thank you for your comment. We included a quality appraisal using the Larg and Moss checklist of cost-of-illness studies. We added information in the Methods section regarding the tool.

1.3 Descriptive Rather Than Analytical Synthesis

The findings are largely descriptive, and no comparative or framework analysis is provided to explain how inequalities affect treatment costs.

We have restructured the discussion to make explicit a conceptual framework comprising two hypotheses: foregone care and accumulated burden, that explain how inequalities affect T2DM treatment costs, moderated by healthcare system structure. While this framework was implicit in our original discussion, we have now named it explicitly, labelled each hypothesis clearly, and added a sentence at the end of each SDH subsection mapping findings onto the framework. We have also added a conceptual diagram illustrating the relationship between SDH, healthcare system type, and cost outcomes.

Limited Novelty and Added Value

The novelty of the study compared to previous reviews is not clearly stated, and many of the results are replications of well-known findings in the existing literature.

We have strengthened the novelty statement in two places. In the introduction, we now state that this is, to the best of our knowledge, the first systematic review to synthesise economic costs of T2DM by SDH, and clarify that cost patterns represent a distinct and policy-relevant dimension of inequality that does not necessarily mirror clinical findings. We also distinguish our contribution from prior reviews on SDH and T2DM outcomes, which have focused on prevalence, complications, and glycaemic control rather than costs.

While individual findings replicate well-known associations between SDH and health outcomes, the contribution of this review lies not in the direction of individual associations which may be anticipated but in the findings that the direction of cost inequality is moderated by healthcare system structure; the foregone care and accumulated burden hypotheses that explain this moderation; and the documentation of geographic gaps in cost research by SDH. We have made these contributions more explicit throughout the discussion.

Policy and Practical Implications

The policy and practical implications are stated in general terms and do not lead to specific, actionable

Thank you for highlighting this. We have now included policy and practical implications in the discussion.

Reviewer #2:

Dear Editor

First off, the authors did a great job setting the stage. The introduction clearly lays out why this is such an important question—type 2 diabetes is a massive global problem, and we know social factors play a huge role in who gets it and how they fare. But this review smartly focuses on a less-discussed angle: the actual financial cost of those inequalities. Their methods are top-notch too; they registered the study on PROSPERO, followed PRISMA guidelines for transparency, and searched a wide range of databases, which gives me a lot of confidence that they found the relevant research out there. The way they've presented all the data in the tables and figures is also really clear and helps you see the patterns at a glance.

Thank you for your kind comments.

Now, where things get a bit tricky, and this is a common challenge in this type of research, is the sheer variety of how different studies define and measure things like 'education' or 'income.' This makes it hard to compare apples to apples, which is why they couldn't do a statistical meta-analysis and had to describe the results narratively. It's not the authors' fault at all; it's just the state of the literature, but it's a significant limitation that means we have to interpret the findings with a bit of caution. I also noticed they pulled a lot of data from the US and China, which is great, but it makes me wonder how these findings apply to places in Africa or South America, which are barely represented. The story of cost inequality might be very different there. And while they are clearly experts at analyzing the numbers, I felt the discussion could have gone a step further to really hammer home the 'so what?' for policymakers. For example, they highlight that in countries where people pay for care out-of-pocket, the poor and rural populations often spend less on treatment, likely because they simply can't afford it and may delay care. But in countries with universal healthcare, the pattern flips, and sometimes the less educated or those facing other disadvantages end up costing the system more, perhaps because of lower health literacy leading to more expensive emergency room visits down the line. That's a crucial insight, and the paper could have ended by more forcefully suggesting what this means for real-world policy—like the need for better health education programs or subsidized transportation for rural patients.

Thank you for pointing this out. As suggested, we have enhanced the discussion section. First by interpreting the results in light of two proposed hypothesis and by expanding the policy implications of the findings including the need for better health education and subsidized transportation.

But all in all, this is a high-quality review. It's rigorous, it's relevant, and it shines a light on a really important issue that doesn't get enough attention. My final judgment is that this is a valuable and well-executed study that adds a lot to our understanding of the economics of diabetes. It's a professional, expert piece of work, no doubt about it, and any researcher or policymaker in this field would benefit from reading it.

Thank you for your comments.

1. Title and Abstract

• Could explicitly state the primary outcome (e.g., "cost differences by SDH") in the abstract.

We have modified the abstract to state the primary outcome as suggested.

• "Cost of inequalities" is slightly ambiguous; "economic burden of health disparities" might be clearer.

We have modified the title as suggested.

2. Introduction

• Overly broad definition of SDH (e.g., includes insurance, which is more of a healthcare access factor).

As suggested, we removed insurance as one of the analysed SDH and now align with the WHO SDH framework.

• Could better distinguish biological sex from gender to justify exclusion.

On page 5, we have expanded the explanation of gender vs sex differences.

3. Methods

• SDH Operationalization: Inconsistent definitions (e.g., "ethnicity" vs. "race").

Thank you for your comment. We have acknowledged the differences between race and ethnicities in the methods section. Unfortunately, most of the included studies use both terms interchangeably, where studies explicitly used racial rather than ethnic categories, we retain the original authors’ terminology.

• Risk of Bias: No assessment for publication bias (e.g., funnel plots).

Thank you for the suggestion. Unfortunately, given the high heterogeneity of the studies retrieved, a meta-analysis was not feasible. Therefore, no effect sizes for each independent study could be calculated and no publication bias assessment is feasible.

• Data Synthesis: Percentage differences are useful but limit comparability due to heterogeneous SDH categorizations.

We acknowledge this limitation. We added a paragraph making this clear in the methods section, as well as in the discussion section.

4. Results

• Heterogeneity: High variability in cost components (e.g., "indirect costs" defined inconsistently) impedes synthesis. Statistical Reporting: Some studies lack adjusted p-values (e.g., Figure 2), weakening causal inference. OOP Costs: Underreported despite relevance in low/middle-income countries (LMICs).

We acknowledge all the points raised by the reviewer as limitations. We have included paragraphs in the discussion explicitly acknowledging this. Documenting the extent of methodological inconsistency in T2DM cost-of-illness research stratified by SDH is in itself a finding and suggestions to improve future studies are now provided in the discussion section.

5. Discussion

• Policy Implications: Underdeveloped. Could suggest actionable strategies (e.g., insurance expansions, rural infrastructure).

Thank you for highlighting the need for enhanced policy implications. We have now added actionable for strategies according to our findings in the discussion section.

• Generalizability: Overreliance on US/China data (13/22 studies) limits global applicability.

Thank you for your comment. Our interpretation was more focused on healthcare models rather than geography. The US and China represent contrasting models within our framework, the US being a mixed, insurance-based system while China represents a system in active transition toward universal coverage but with persistent OOP burden, particularly in rural areas. The contrast between these two contexts allowed us to observe that the direction of cost inequality by SDH appears to be moderated by healthcare financing structure rather than being a fixed, universal pattern. We suggest that the generalizability of our findings should be considered less in terms of geographic representativeness and more in terms of the range of healthcare financing models represented. Nonetheless, we agree this is an important limitation and have strengthened our limitations section to explicitly recommend that future studies from underrepresented regions are needed to test whether the conceptual framework proposed in this review holds in those contexts.

• Gender Exclusion: Justification is weak; gender influences health-seeking behavior and costs.

This is correct. However, in the studies included, gender is not operationalised as a the socially constructed characteristics rather than sex. We have included a more ample explanation of this in the methods section as well as in the discussions section calling for a proper categorisation of gender.

6. Conclusion

• Lacks forward-looking recommendations (e.g., "future studies should...").

We have now included a paragraph about future studies, establishing the main gaps the review found.

• Does not reiterate the public health significance of addressing cost inequalities.

In the discussion section, we have added a closing statement to the conclusion explicitly reiterating the public health significance of addressing cost inequalities in T2DM treatment.

7. Overall Assessment

• Heterogeneity: Limits quantitative synthesis; meta-analysis was infeasible.

This is correct. We state in the paper that meta-analysis was not feasible.

• Geographic Bias: Underrepresentation of Africa/Latin America.

This limitation is very concerning given the inequalities observed in these regions. We clearly state the need for research in these regions

• SDH Scope: Some factors (e.g., insurance) may not align with WHO SDH framework.

We have aligned our definition with the WHO SDH framework and as suggested removed insurance as one of the SDHs analysed.

8. Recommendations for Improvement

1. Clarify SDH Definitions: Align with WHO SDH framework (e.g., exclude insurance).

We have aligned our definition with the WHO SDH framework and as suggested removed insurance as one of the SDHs analysed.

2. Enhance Statistical Synthesis: Use random-effects models where possible; report confidence intervals.

Thank you for the suggestion. Random-effects meta-analysis requires pooled effect estimates with extractable standard errors across comparable outcomes. Given the substantial heterogeneity in SDH definitions, cost components, currencies, healthcare systems, and study designs across included studies, a pooled estimate would not be suitable. Regarding confidence intervals, the majority of included studies report group means alongside standard deviations or standard errors, but do not report variance measures for the difference between groups. Additionally, we were concerned that presenting confidence intervals alongside percentage differences could imply a level of cross-study comparability that our narrative and thematic synthesis approach does not claim nor support. We have retained percentage differences as a descriptive measure of the direction and approximate magnitude of cost gradients within each study, accompanied by significance levels as originally reported (crude and/or adjusted, as available). We believe this approach, combined with the thematic synthesis and conceptual framework added in response to other reviewer comments, provides a transparent and methodologically suitable synthesis given the nature of the available evidence.

3. Expand Policy Discussion: Propose interventions (e.g., subsidized care for low-income groups).

We have added policy recommendations in the discussion section. Thank you for your suggestion.

4. Address Gaps: Include gender, LMICs beyond Asia, and standardized cost components.

Regarding gender, we refer to our methods section, where we explain that gender was excluded as an SDH because it is rarely operationalised as such in the cost literature — studies predominantly report biological sex rather than gender identity or roles — and because biological sex, while relevant to T2DM prevalence, represents a biomedical rather than social determinant and falls outside the SDH framework as defined for this review.

Regarding LMICs, we have expanded our discussion of healthcare financing models to address this, noting that countries such as Bangladesh, India, and Pakistan represent out-of-pocket-dominant systems, and have discussed the underreporting of OOP costs specifically in these contexts as both a finding and a limitation of the available evidence base. We have also added a recommendation in our conclusion for future studies from underrepresented LMIC regions, particularly Africa and Latin America.

Regarding standardised cost components, we have addressed this in our discussion of heterogeneity, noting the inconsistent classification of cost components across studies (e.g. transportation costs classified as direct non-medical in some studies and indirect in others) and recommending that future cost-of-illness studies adopt standardised costing methods to improve comparability.

9. Final Judgment

This is a high-quality systematic review with methodological rigor and significant contributions to understanding diabetes cost inequalities. minor Revision to address critiques would strengthen its impact.

Thank you for your comments and very insightful suggestions.

Reviewer #3: The authors conducted a rigorous systematic review including updates from H2 of 2025. As noted in the above section, the review has been performed appropriately and rigorously, and the underlying data/references are fully transparent. The topic is of interest, and the authors did a thorough job vetting of the literature.

Thank you for your comments.

However, the discussion and how the authors deal with the question of cost of inequality in the treatment of Type 2 Diabetes can be better fleshed out. Part of the issue is the combining of a number of disparate countries into one review. I would recommend in the discussion potentially discussing the different determinants by region - i.e. US, Asia, Europe and Latin America and come up with conclusions by region as well as by factor (i.e. income, etc).

Than

# Peer review: Decision Letter 1

**DOI:** 10.1371/journal.pone.0354198.r003

**Authors:** Pasyodun Koralage Buddhika Mahesh

6 Jul 2026

The economic burden of Type 2 Diabetes by social determinants of health: A systematic review

PONE-D-25-68922R1

Dear Dr. Alarcón-Yaquetto,

We’re pleased to inform you that your manuscript has been judged scientifically suitable for publication and will be formally accepted for publication once it meets all outstanding technical requirements.

Within one week, you’ll receive an e-mail detailing the required amendments. When these have been addressed, you’ll receive a formal acceptance letter and your manuscript will be scheduled for publication.

An invoice will be generated when your article is formally accepted. Please note, if your institution has a publishing partnership with PLOS and your article meets the relevant criteria, all or part of your publication costs will be covered. Please make sure your user information is up-to-date by logging into Editorial Manager at Editorial Manager® and clicking the ‘Update My Information' link at the top of the page. For questions related to billing, please contact billing support.

If your institution or institutions have a press office, please notify them about your upcoming paper to help maximize its impact. If they’ll be preparing press materials, please inform our press team as soon as possible -- no later than 48 hours after receiving the formal acceptance. Your manuscript will remain under strict press embargo until 2 pm Eastern Time on the date of publication. For more information, please contact onepress@plos.org.

Kind regards,

Pasyodun Koralage Buddhika Mahesh

Academic Editor

PLOS One

Additional Editor Comments (optional):

Reviewers' comments:

Reviewer's Responses to Questions

**Comments to the Author**

1. If the authors have adequately addressed your comments raised in a previous round of review and you feel that this manuscript is now acceptable for publication, you may indicate that here to bypass the “Comments to the Author” section, enter your conflict of interest statement in the “Confidential to Editor” section, and submit your "Accept" recommendation.

Reviewer #3: All comments have been addressed

Reviewer #4: (No Response)

**********

2. Is the manuscript technically sound, and do the data support the conclusions?

The manuscript must describe a technically sound piece of scientific research with data that supports the conclusions. Experiments must have been conducted rigorously, with appropriate controls, replication, and sample sizes. The conclusions must be drawn appropriately based on the data presented.

Reviewer #3: Yes

Reviewer #4: (No Response)

**********

3. Has the statistical analysis been performed appropriately and rigorously?

Reviewer #3: Yes

Reviewer #4: (No Response)

**********

4. Have the authors made all data underlying the findings in their manuscript fully available?

The PLOS Data policy requires authors to make all data underlying the findings described in their manuscript fully available without restriction, with rare exception (please refer to the Data Availability Statement in the manuscript PDF file). The data should be provided as part of the manuscript or its supporting information, or deposited to a public repository. For example, in addition to summary statistics, the data points behind means, medians and variance measures should be available. If there are restrictions on publicly sharing data—e.g. participant privacy or use of data from a third party—those must be specified.

Reviewer #3: Yes

Reviewer #4: (No Response)

**********

5. Is the manuscript presented in an intelligible fashion and written in standard English?

PLOS ONE does not copyedit accepted manuscripts, so the language in submitted articles must be clear, correct, and unambiguous. Any typographical or grammatical errors should be corrected at revision, so please note any specific errors here.

Reviewer #3: Yes

Reviewer #4: (No Response)

**********

6. Review Comments to the Author

Please use the space provided to explain your answers to the questions above. You may also include additional comments for the author, including concerns about dual publication, research ethics, or publication ethics. (Please upload your review as an attachment if it exceeds 20,000 characters)

Reviewer #3: Thank you for revising the manuscript to incorporate my comments. I believe the manuscript reads better and will help contribute to the literature.

Reviewer #4: The corrections were satisfying. The manuscript is acceptable for publication. Best wishes for the authors.

**********

7. PLOS authors have the option to publish the peer review history of their article (what does this mean?). If published, this will include your full peer review and any attached files.

If you choose “no”, your identity will remain anonymous but your review may still be made public.

**Do you want your identity to be public for this peer review?**  For information about this choice, including consent withdrawal, please see our Privacy Policy.

Reviewer #3: **Yes:**  Jamie Partridge Grossman

Reviewer #4: **Yes:**  Seyyed Amir Yasin Ahmadi

**********

# Editor report: Acceptance letter

**DOI:** 10.1371/journal.pone.0354198.r004

**Authors:** Pasyodun Koralage Buddhika Mahesh

PONE-D-25-68922R1

PLOS One

Dear Dr. Alarcón-Yaquetto,

I'm pleased to inform you that your manuscript has been deemed suitable for publication in PLOS One. Congratulations! Your manuscript is now being handed over to our production team.

At this stage, our production department will prepare your paper for publication. This includes ensuring the following:

* All references, tables, and figures are properly cited

* All relevant supporting information is included in the manuscript submission,

* There are no issues that prevent the paper from being properly typeset

You will receive further instructions from the production team, including instructions on how to review your proof when it is ready. Please keep in mind that we are working through a large volume of accepted articles, so please give us a few days to review your paper and let you know the next and final steps.

Lastly, if your institution or institutions have a press office, please let them know about your upcoming paper now to help maximize its impact. If they'll be preparing press materials, please inform our press team within the next 48 hours. Your manuscript will remain under strict press embargo until 2 pm Eastern Time on the date of publication. For more information, please contact onepress@plos.org.

You will receive an invoice from PLOS for your publication fee after your manuscript has reached the completed accept phase. If you receive an email requesting payment before acceptance or for any other service, this may be a phishing scheme. Learn how to identify phishing emails and protect your accounts at https://explore.plos.org/phishing.

If we can help with anything else, please email us at customercare@plos.org.

Thank you for submitting your work to PLOS ONE and supporting open access.

Kind regards,

PLOS ONE Editorial Office Staff

on behalf of

Dr. Pasyodun Koralage Buddhika Mahesh

Academic Editor

PLOS One
